# COL10A1^+^ fibroblasts promote colorectal cancer metastasis and M2 macrophage polarization with pan-cancer relevance

**DOI:** 10.1186/s13046-025-03510-8

**Published:** 2025-08-18

**Authors:** Shangshang Hu, Muzi Ding, Jinwei Lou, Jian Qin, Yuhan Chen, Zixuan Liu, Yue Li, Junjie Nie, Mu Xu, Huiling Sun, Xinliang Gu, Tao Xu, Shukui Wang, Shukui Wang, Yuqin Pan

**Affiliations:** 1 General Clinical Research Center, Nanjing First Hospital, Nanjing Medical University, Nanjing, 210006 Jiangsu China; 2 School of Medicine, Southeast University, Nanjing, 210009 Jiangsu China; 3 Jiangsu Collaborative Innovation Center on Cancer Personalized Medicine, Nanjing Medical University, Nanjing, 211100 Jiangsu China; 4 School of Basic Medicine and Clinical Pharmacy, China Pharmaceutical University, Nanjing, 211122 Jiangsu China; 5 Nanjing Medical Key Laboratory of Laboratory Diagnostics, Nanjing, China

**Keywords:** Cancer-associated fibroblasts, COL10A1, Colorectal cancer, Pan-cancer

## Abstract

**Background:**

Colorectal cancer (CRC) is a common gastrointestinal cancer with poor response to therapy and high metastatic risk. Cancer-associated fibroblasts (CAFs) support tumor progression, but their functional heterogeneity remains poorly understood.

**Methods:**

We integrated multi-omics data from 10,164 samples, including single-cell, bulk, spatial transcriptomics, and proteomics, to identify and characterize CAF subpopulations. Functional validation was performed using molecular assays, in vivo models, and drug screening.

**Results:**

We identified a COL10A1-positive fibroblast subpopulation (COL10A1^+^Fib) associated with CRC progression and poor patient prognosis. COL10A1^+^Fib promotes tumor cell proliferation, immune suppression, and metastasis. Mechanistically, COL10A1^+^Fib facilitates epithelial–mesenchymal transition (EMT) in CRC cells via COL10A1 secretion and induces M2 macrophage polarization through the COL10A1/CD18/JAK1/STAT3 signaling axis. In turn, M2 macrophages enhance COL10A1 expression in fibroblasts via the TGF-β/RUNX2 pathway, forming a pro-tumorigenic feedback loop. The DNA-PKcs inhibitor NU7441 reduces COL10A1 expression, suppresses CAF activity, and reverses EMT and M2 polarization. Pan-cancer analysis suggests that COL10A1^+^Fib may have similar functional roles across multiple major solid tumors.

**Conclusion:**

Our study identifies a CAF subpopulation, COL10A1^+^Fib, associated with CRC progression and immune suppression, suggesting it as a potential therapeutic target in CRC and possibly other malignancies.

**Supplementary Information:**

The online version contains supplementary material available at 10.1186/s13046-025-03510-8.

## Graphical Abstract

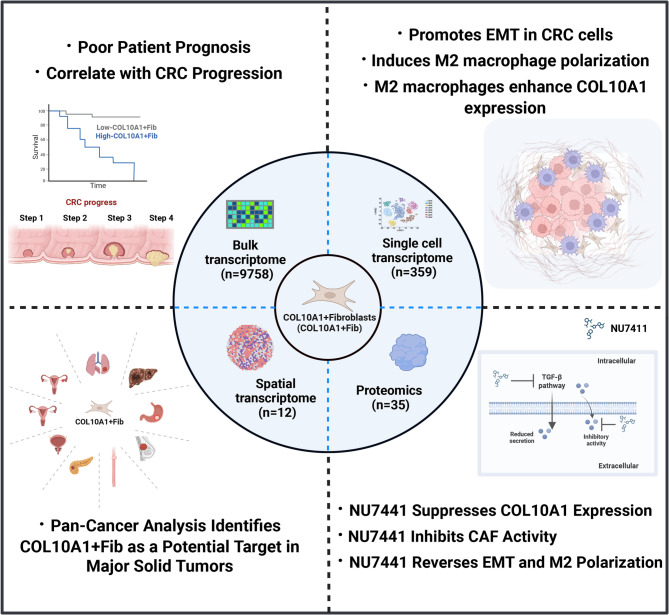

## Introduction

Colorectal cancer (CRC) is among the most prevalent malignancies worldwide and remains a leading cause of cancer-related morbidity and mortality [1]. Despite continuous advances in diagnosis and treatment, the long-term survival of CRC patients remains limited due to late-stage metastasis and immune evasion [2, 3]. Given the incomplete understanding of CRC progression mechanisms, identifying key drivers within the tumor microenvironment (TME), especially those originating from non-cancerous stromal components, has become a critical focus of translational CRC research.

Within the complex TME, cancer-associated fibroblasts (CAFs) represent the dominant stromal cell population of non-tumor origin and are deeply involved in cancer initiation, progression, invasion, and immune regulation [4, 5]. Through the secretion of cytokines, extracellular matrix (ECM) proteins, and signaling molecules, CAFs contribute to microenvironment remodeling, immune evasion, and angiogenesis, thereby promoting tumor growth and metastasis [6]. Advances in single-cell and spatial transcriptomic technologies have revealed that CAFs are not a uniform population but instead comprise multiple functionally heterogeneous subtypes, including myCAF, iCAF, and apCAF, with distinct spatial distributions and context-dependent roles across cancer types and stages [7, 8]. While substantial progress has been made in characterizing CAF subsets in breast and pancreatic cancers, a systematic classification and mechanistic understanding of CAF subtypes in CRC remains lacking [9, 10]. Deciphering the functional heterogeneity of CAFs in CRC is essential for identifying their pathogenic contributions and potential therapeutic vulnerabilities.

Collagen type X alpha-1 (COL10A1) is a short-chain fibrillar collagen originally identified in hypertrophic chondrocytes and classically associated with cartilage maturation and skeletal development [11]. Recent multi-omics studies have shown aberrant upregulation of COL10A1 in the tumor stroma of breast, pancreatic, and gastric cancers, where it correlates with lymph node metastasis, hematogenous spread, and poor prognosis [12, 13]. Functionally, COL10A1 contributes to ECM remodeling and can interact with proteins such as prolyl 4-hydroxylase subunit beta (P4HB) and integrin subunit beta 1 (ITGB1) to facilitate tumor cell proliferation and metastasis [14, 15]. However, the precise origin, cellular sources, and mechanistic role of COL10A1 within the CRC TME remain unclear, as does its regulatory network and druggability.

In this study, we integrated multi-omics data (bulk RNA-seq, single-cell, spatial transcriptomics, and proteomics) with in vitro and in vivo experiments to identify and characterize the COL10A1^+^ fibroblast subpopulation (COL10A1^+^Fib) in CRC. We observed that COL10A1^+^Fib is associated with tumor metastasis and the promotion of an immunosuppressive microenvironment. Additionally, we identified NU7441 as a small-molecule inhibitor that attenuates COL10A1^+^Fib activity and its tumor-promoting effects. Pan-cancer analyses suggest that COL10A1^+^Fib is enriched in several solid tumors, highlighting its potential as a therapeutic target. Our findings contribute to understanding CAF heterogeneity and provide potential avenues for the development of anti-stromal strategies in cancer therapy.

## Materials and methods

### CRC specimens and data collection

A total of 35 paired tumor and adjacent non-tumor tissue samples were collected from CRC patients at the First Affiliated Hospital of Nanjing Medical University (clinical details provided in Supplementary Table 1). All human tissue sample collection and usage procedures were approved by the Ethics Committee of the First Affiliated Hospital of Nanjing Medical University. Informed consent was obtained from all participants, and the study adhered to the principles of the Declaration of Helsinki and relevant ethical guidelines. This study systematically integrated multi-omics data from public databases, including published literature, The Cancer Genome Atlas (TCGA), the Genotype-Tissue Expression (GTEx) project, Gene Expression Omnibus (GEO), ArrayExpress, and the Spatial Transcript Omics DataBase (STOmics DB). In total, 3,395 CRC-related samples were included, comprising 3,122 bulk RNA-seq samples, 234 single-cell RNA-seq samples, and 4 spatial transcriptomic samples. Additionally, multi-omics data were collected for nine other high-mortality solid tumor types: lung, liver, stomach, breast, esophagus, pancreas, prostate, cervix, and ovary. These datasets encompassed 6,769 samples in total, including 6,636 bulk RNA-seq samples, 125 single-cell RNA-seq samples, and 8 spatial transcriptomic samples. Detailed information regarding the pan-cancer datasets is provided in Supplementary Tables 2 and 3.

### Bulk RNA-seq data processing and integration

To minimize batch effects among CRC datasets generated using the same sequencing platform, batch correction and data normalization were performed using the “sva” R package. CRC molecular subtyping was conducted using the “Lothelab/CMScaller” R package, classifying TCGA-CRC samples into four consensus molecular subtypes (CMS1–CMS4) as previously described [16].

### Single-cell RNA-Seq data processing

The ScRNA data (human and mouse data) were processed using the “Seurat” R package. The analysis methods and parameters for both human and mouse single-cell data were consistent. Low-quality cells were filtered based on the following criteria: fewer than 200 or more than 5,000 detected features, or > 20% mitochondrial gene expression. Principal component analysis (PCA) was performed using the “RunPCA” function, followed by dimensionality reduction and clustering using “RunUMAP” and “RunTSNE” functions. Cell-type-specific marker genes were identified for each cluster using the “FindAllMarkers” function with parameters set to min.pct = 0.25 and logfc.threshold > 0.25. Data integration and batch effect correction across samples were performed using the “harmony” R package [17]. Cell type annotation was based on the “SingleR” R package and the CellMarker 2.0 database [18, 19]. To distinguish malignant from non-malignant epithelial cells, copy number variation (CNV) inference was conducted using the “intercnv” R package, with normal epithelial cells used as the reference baseline (parameters: cutoff = 0.1, HMM = FALSE) [20].

### Spatial transcriptomic data processing and spatial mapping

Spatial transcriptomic (ST) data were preprocessed using standard workflows in the “Seurat” R package, including dimensionality reduction with RunPCA and clustering using “FindNeighbors” and “FindClusters”. Spatial mapping between ST and single-cell datasets was performed using the “CellTrek” R package [21] to determine the spatial localization of specific cell types within tissue sections. Malignant tumor cell identification and spatial gene scoring were performed using the “SpaCET” R package to support spatial annotation and heterogeneity analysis [22]. Ligand–receptor spatial colocalization analysis was conducted using the “SpaGene” R package [23].

### Single-sample gene set enrichment analysis (ssGSEA)

Signature scores were generated with the GSVA R package (method = “ssgsea”). Differentially expressed genes identified from our scRNA-seq dataset (avg_log_2_FC > 1, p_val_adj < 0.05) were compiled into cell-subtype–specific gene sets. Bulk RNA-seq datasets (TCGA, GEO) were converted to log_2_-transformed TPM values, and scRNA/spatial transcriptomics data were normalized with SCTransform. Scores were computed by calling gsva(expr, geneSet, method = “ssgsea”, kcdf = “Gaussian”, abs.ranking = TRUE) and rescaled to a 0–1 range for each sample or spatial spot. All marker genes used are provided in Supplementary Table 4.

### Statistical analysis

All statistical analyses were performed using GraphPad Prism 9.0 (GraphPad Software, San Diego, CA, USA) and R software (version 4.3.0). Correlations between continuous variables were assessed using Spearman’s rank correlation test. For comparisons between two groups, two-tailed Student’s t-test was used for normally distributed data; otherwise, the non-parametric Wilcoxon rank-sum test (Mann–Whitney U test) was applied. For multiple group comparisons, appropriate non-parametric tests were selected based on data distribution. All in vitro functional assays were independently repeated at least three times under identical conditions, and data are presented as mean ± standard deviation (SD). A *P* value < 0.05 was considered statistically significant. Significance levels are indicated in figures and results as follows: *P < 0.05; **P < 0.01; ***P < 0.001; ****P < 0.0001.

Other materials and methods are provided in the Supplementary Methods file.

## Result

### Fibroblasts display Gene-Expression signatures linked to metastasis and immune suppression in advanced CRC

**Fig. 1:**
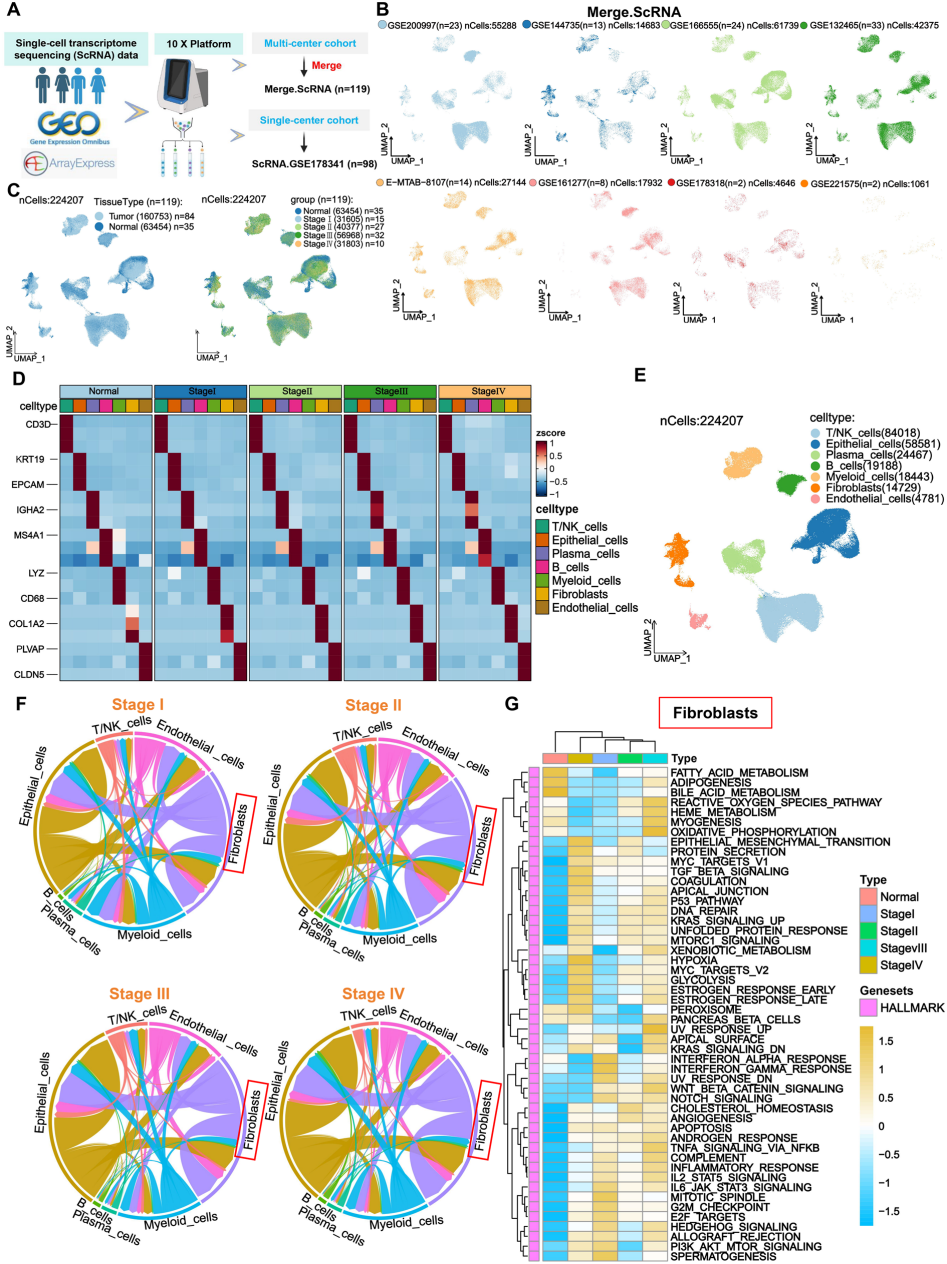
Fibroblasts Exhibit Pro-Metastatic and Immunosuppressive Features in Advanced CRC Based on Merge.ScRNA Data. **A**. scRNA-seq data from GEO and ArrayExpress were integrated into two cohorts: the multi-center cohort (Merge.ScRNA) and single-center cohort (ScRNA.GSE178341). **B**. UMAP distribution of single cells from the eight datasets within Merge.ScRNA. **C**. UMAP plot of Merge.ScRNA data stratified by tissue type and TNM stage. **D**. Heatmap showing expression of canonical surface markers across cell types in different TNM stages. **E**. UMAP clustering of seven major cell types. **F**. Circular plot showing intercellular communication strength and weights across cell types. **G**. Heatmap of GSVA pathway enrichment in fibroblasts across TNM stages

To investigate the role of fibroblasts in CRC progression, we curated publicly available scRNA datasets from the GEO and ArrayExpress databases, constructing two independent cohorts: a multi-center cohort (Merge.ScRNA, *n* = 119) composed of eight datasets, and a single-center cohort (ScRNA.GSE178341, *n* = 98) derived from a single dataset (Fig. 1A). After stringent quality control, 224,207 high-quality cells were retained in the Merge.ScRNA cohort. Cellular distribution maps were constructed based on dataset origin, tissue type, and TNM staging (Fig. 1B–C). Seven major cell types were annotated using canonical surface markers, including T/NK cells (CD3D), epithelial cells (KRT19), plasma cells (IGHA2), B cells (MS4A1), myeloid cells (LYZ), fibroblasts (COL1A1), and endothelial cells (PLVAP) (Fig. 1D–E). Cell–cell communication analysis revealed that fibroblasts exhibited the second-highest interaction strength after epithelial tumor cells across all TNM stages, suggesting their potential role in CRC progression (Fig. 1F). Gene Set Variation Analysis (GSVA) revealed that fibroblasts in TNM stage IV samples were markedly enriched in pathways associated with metastasis and immune suppression, such as epithelial–mesenchymal transition (EMT), TGF-β signaling, and hypoxia. (Fig. 1G). These findings were validated in the independent ScRNA.GSE178341 cohort, reinforcing the biological significance of fibroblasts in advanced CRC and providing a foundation for downstream fibroblast subpopulation analysis (Supplementary Fig. 1A–E).

### Identification of COL10A1^+^Fib as associated with CRC progression

**Fig. 2:**
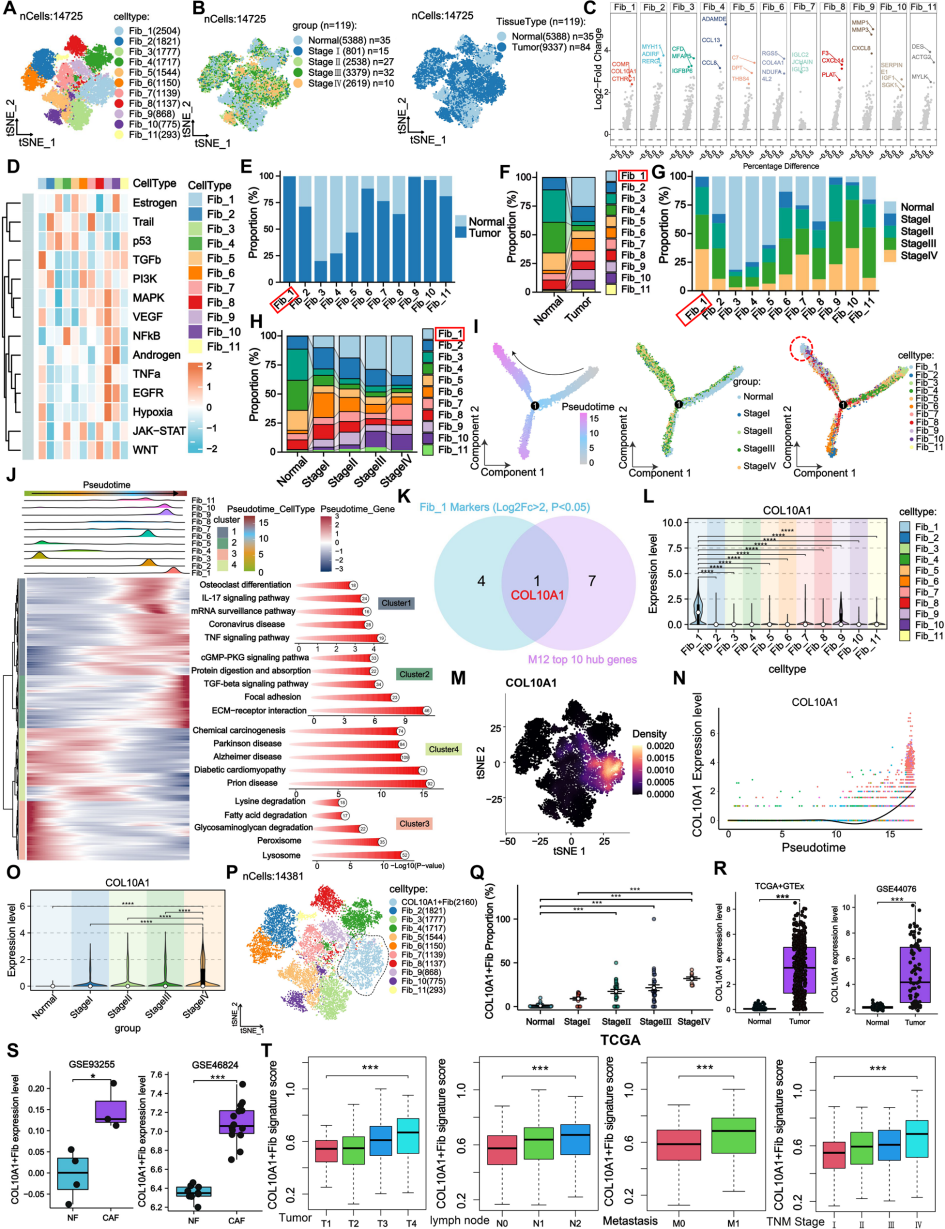
Identification of COL10A1^+^Fib as associated with CRC progression. **A**. tSNE plot of 11 fibroblast subclusters (Fib_1–Fib_11). **B**. tSNE plots showing subcluster distribution across tissue types and TNM stages. **C**. Volcano plots of the top three marker genes for each subcluster. **D**. Heatmap of functional pathway enrichment among the 11 fibroblast subclusters. **E**–**F**. Comparison of subcluster proportions between normal and tumor tissues. **G**–**H**. Proportional distribution of subclusters across TNM stages. **I**. Pseudotime trajectory analysis indicating Fib_1 as a terminally differentiated subcluster. **J**. Enrichment of pseudotime modules (Cluster1–4) in key biological processes. **K**. Venn diagram identifying COL10A1 as the intersection of highly expressed genes in Fib_1 and top hub genes in the M12 module. **L**–**M**. Expression distribution of COL10A1 across fibroblast subclusters (violin plot and tSNE). **N**. Pseudotime expression dynamics of COL10A1. **O**. COL10A1 expression stratified by TNM stage. **P**. Definition of COL10A1^+^Fib by excluding COL10A1⁻ cells from Fib_1. **Q**. Proportional changes in COL10A1^+^Fib across different TNM stages in the Merge.ScRNA. **R**. COL10A1 expression levels in CRC tissues versus normal tissues based on bulk datasets (TCGA + GTEx and GSE44076). **S**. Comparative expression of COL10A1^+^Fib between CAFs and NFs (datasets: GSE93255 and GSE46824). **T**. Infiltration levels of COL10A1^+^Fib across different clinicopathological subgroups in the TCGA CRC cohort

To further dissect fibroblast heterogeneity during CRC progression, fibroblasts from the Merge.ScRNA cohort were re-clustered, yielding 11 distinct subpopulations (Fib_1–Fib_11) (Fig. 2A). A tSNE map was generated to visualize their distribution across different tissue sources and TNM stages (Fig. 2B). All subclusters expressed canonical fibroblast markers (COL1A1, COL1A2, COL3A1), confirming the clustering accuracy (Supplementary Fig. 2A) The top three marker genes of each subcluster were identified (Fig. 2C, Supplementary Fig. 2B), and functional enrichment analysis revealed distinct pathway activation profiles among subclusters (Fig. 2D). Cellular proportion analysis showed that Fib_1 was highly enriched in tumor tissues and progressively increased in frequency with advancing TNM stage (Fig. 2E–H), suggesting a potential role in CRC progression. Pseudotime trajectory analysis indicated that Fib_1 resided at the terminal branch of differentiation (Fig. 2I), and the associated gene module (Cluster2) was enriched in genes related to extracellular matrix remodeling, adhesion, and immunosuppression (Fig. 2J), suggesting a potential tumor-promoting role. Consequently, Fib_1 was selected for further analysis. Using high-dimensional weighted gene co-expression network analysis (hdWGCNA), we constructed a scale-free network for fibroblasts and identified 16 co-expression modules (Supplementary Fig. 2C–E). The M12 module was highly enriched in Fib_1 (Supplementary Fig. 2F–G), and co-expression analysis revealed strong interactions among the top 25 hub genes (Supplementary Fig. 2H). By intersecting genes that were both highly expressed in Fib_1 (logFC > 2, *p* < 0.05) and ranked in the top 10 of the M12 module, COL10A1 was identified as a specific marker (Fig. 2K). COL10A1 expression was significantly higher in Fib_1 compared to other subclusters (Fig. 2L–M) and was markedly upregulated in advanced CRC samples (Fig. 2N–O). Subsequently, non-expressing COL10A1 cells were excluded from Fib_1, and the remaining cells were defined as COL10A1^+^Fib (Fig. 2P). COL10A1^+^Fib were significantly enriched in tumor tissues and their proportion increased progressively with TNM stage (Fig. 2Q). The enrichment of COL10A1^+^Fib in late-stage CRC was independently confirmed in the ScRNA.GSE178341 cohort (Supplementary Fig. 3A–G), supporting its robustness. Bulk transcriptomic data from TCGA + GTEx and GSE44076 confirmed that COL10A1 expression was significantly upregulated in tumor tissues (Fig. 2R), with higher expression in CAFs compared to normal fibroblasts (Fig. 2S). Further stratified analysis revealed elevated COL10A1^+^Fib infiltration in advanced clinical stages, nodal metastasis, and distant metastasis subgroups (Fig. 2T).

### Clinical validation of COL10A1^+^Fib in CRC progression and prognosis

**Fig. 3:**
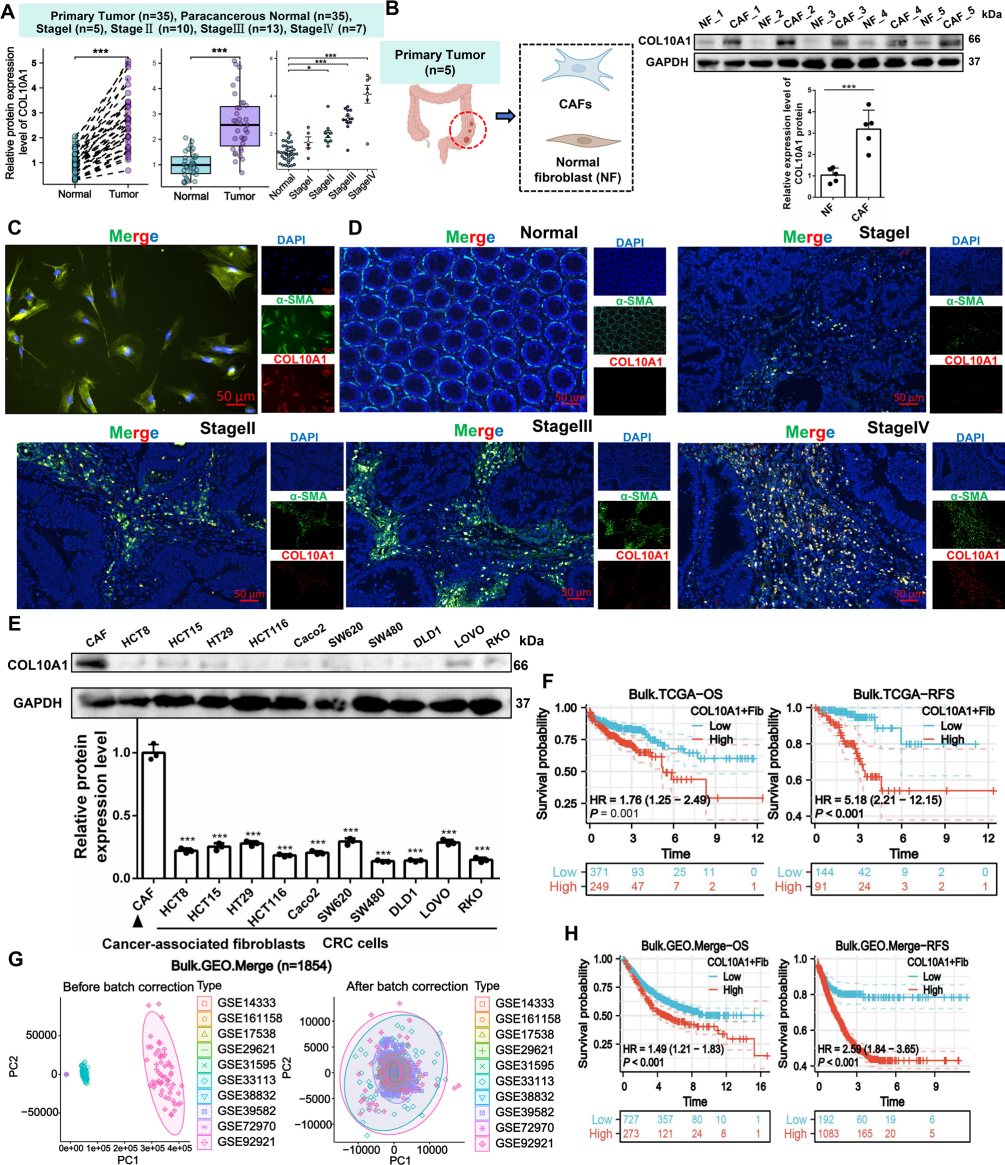
Validation of the association between COL10A1^+^Fib and CRC progression and prognosis in clinical samples. **A**. COL10A1 protein levels in normal vs. tumor tissues and across TNM stages based on in-house CRC samples; Primary Tumor (*n* = 35), Paracancerous Normal (*n* = 35). **B**. Protein-level comparison of COL10A1 expression between isolated CAFs and NFs; Primary Tumor (*n* = 5), Paracancerous Normal (*n* = 5). **C**. Co-expression of COL10A1 and α-SMA in CAFs visualized by immunofluorescence; Primary Tumor (*n* = 5); Scale bars, 50 μm. **D**. Distribution of COL10A1^+^Fib across normal tissues and different TNM stages; Primary Tumor (*n* = 35), Paracancerous Normal (*n* = 35); Scale bars, 50 μm. **E**. COL10A1 protein expression in 10 human CRC cell lines compared to CAFs. **F**. Kaplan–Meier curves for overall survival (OS) and relapse-free survival (RFS) based on COL10A1^+^Fib levels in the TCGA cohort. **G**. Batch-corrected integration of 10 GEO datasets in the Bulk.GEO.Merge cohort. **H**. Survival analysis of COL10A1^+^Fib-high and -low patients in the Bulk.GEO.Merge cohort (OS and RFS). *P < 0.05; ***P < 0.001

At the protein level, examination of 35 paired colorectal cancer clinical specimens showed that COL10A1 was over-expressed in the tumour group in both paired and unpaired analyses, and its expression increased with advancing stage (Fig. 3A). Western blotting in primary cells showed higher COL10A1 levels in CAFs compared to adjacent normal fibroblasts (Fig. 3B), and immunofluorescence staining demonstrated co-expression of COL10A1 and α-SMA in CAFs (Primary Tumor: *n* = 5) (Fig. 3C), with widespread expression in advanced-stage CRC tissues (Paracancerous Normal: *n* = 35, StageI: *n* = 5, StageII: *n* = 10, StageIII: *n* = 13, StageIV: *n* = 7) (Fig. 3D). Comparative analysis of 10 human CRC cell lines and CAFs confirmed that COL10A1 was primarily derived from CAFs (Fig. 3E). Moreover, a Col10a1^+^Fib subpopulation was identified in seven murine single-cell RNA-seq datasets, suggesting conservation across species (Supplementary Fig. 4A–D). In the TCGA cohort, high infiltration of COL10A1^+^Fib was significantly associated with poorer overall survival (OS) and recurrence-free survival (RFS) (Fig. 3F). We also integrated ten GEO datasets with survival information from the GPL570 platform (Bulk.GEO.Merge, *n* = 1854) (Fig. 3G), where high COL10A1^+^Fib infiltration was similarly associated with reduced OS and RFS (Fig. 3H).

**Fig. 4:**
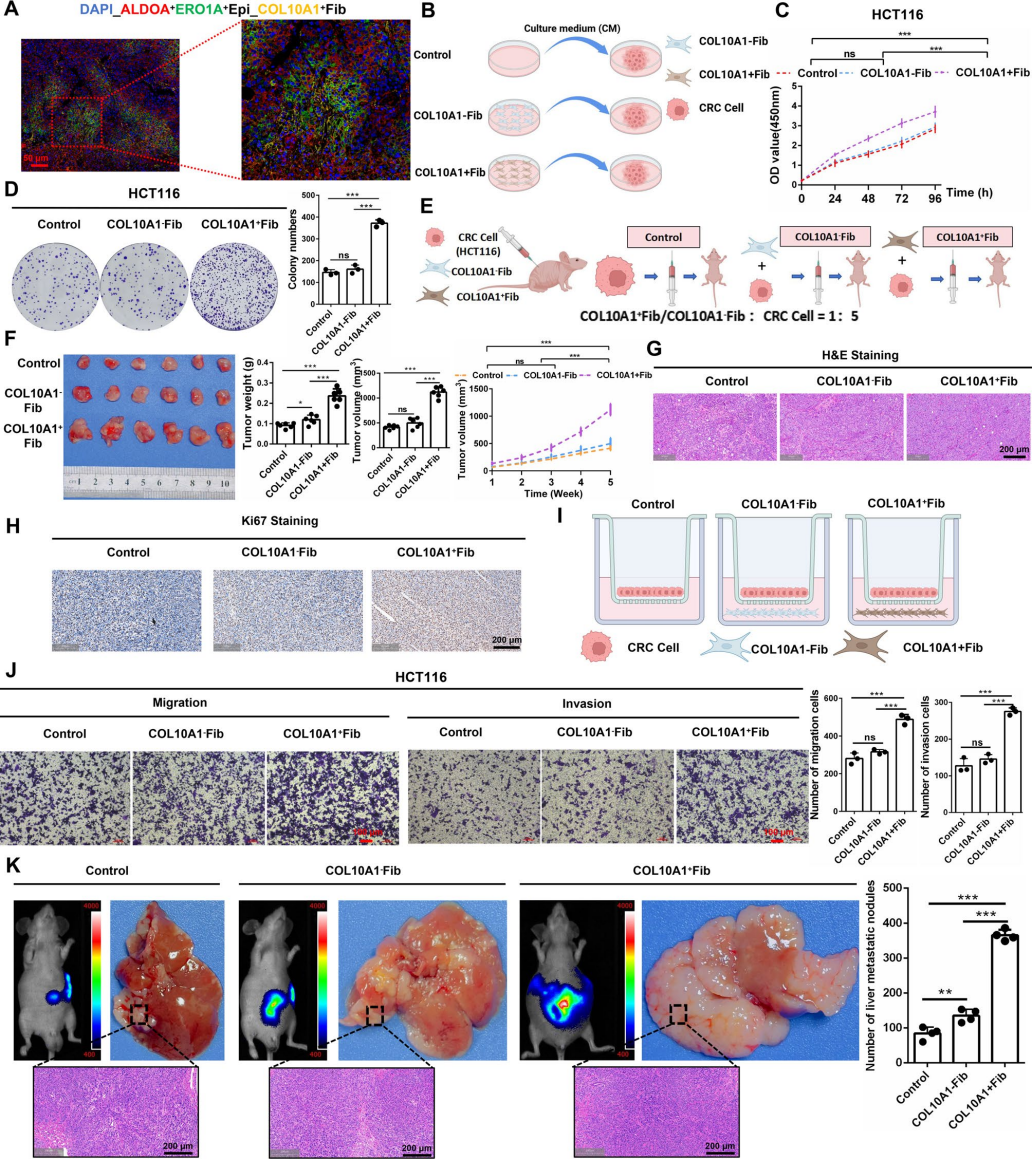
COL10A1^+^Fib promote CRC cell proliferation, invasion, and liver metastasis. **A**. Multiplex immunofluorescence staining shows spatial colocalization of COL10A1^+^Fib with ERO1A^+^/ALDOA^+^ epithelial cells (Epi_1) (Primary Tumor: *n* = 35). **B**. Schematic diagram of the in vitro co-culture system involving CRC cells (HCT116) co-cultured with control fibroblasts, COL10A1⁻Fib, or COL10A1^+^Fib. **C**. CCK-8 assay assessing proliferation of HCT116 cells under different co-culture conditions. **D**. Colony formation assay evaluating the clonogenic capacity of HCT116 cells. **E**. Schematic workflow of the subcutaneous xenograft model in nude mice. **F**. Tumor volume and tumor weight measurements in xenograft-bearing mice across experimental groups. **G**. H&E staining of tumor tissues to assess morphological differences (per group: *n* = 6). Scale bars, 200 μm. **H**. Ki67 immunohistochemistry (IHC) for evaluation of proliferative activity within tumor tissues (per group: *n* = 6). **I**. Schematic representation of the Transwell migration and invasion assay

**Fig. 5:**
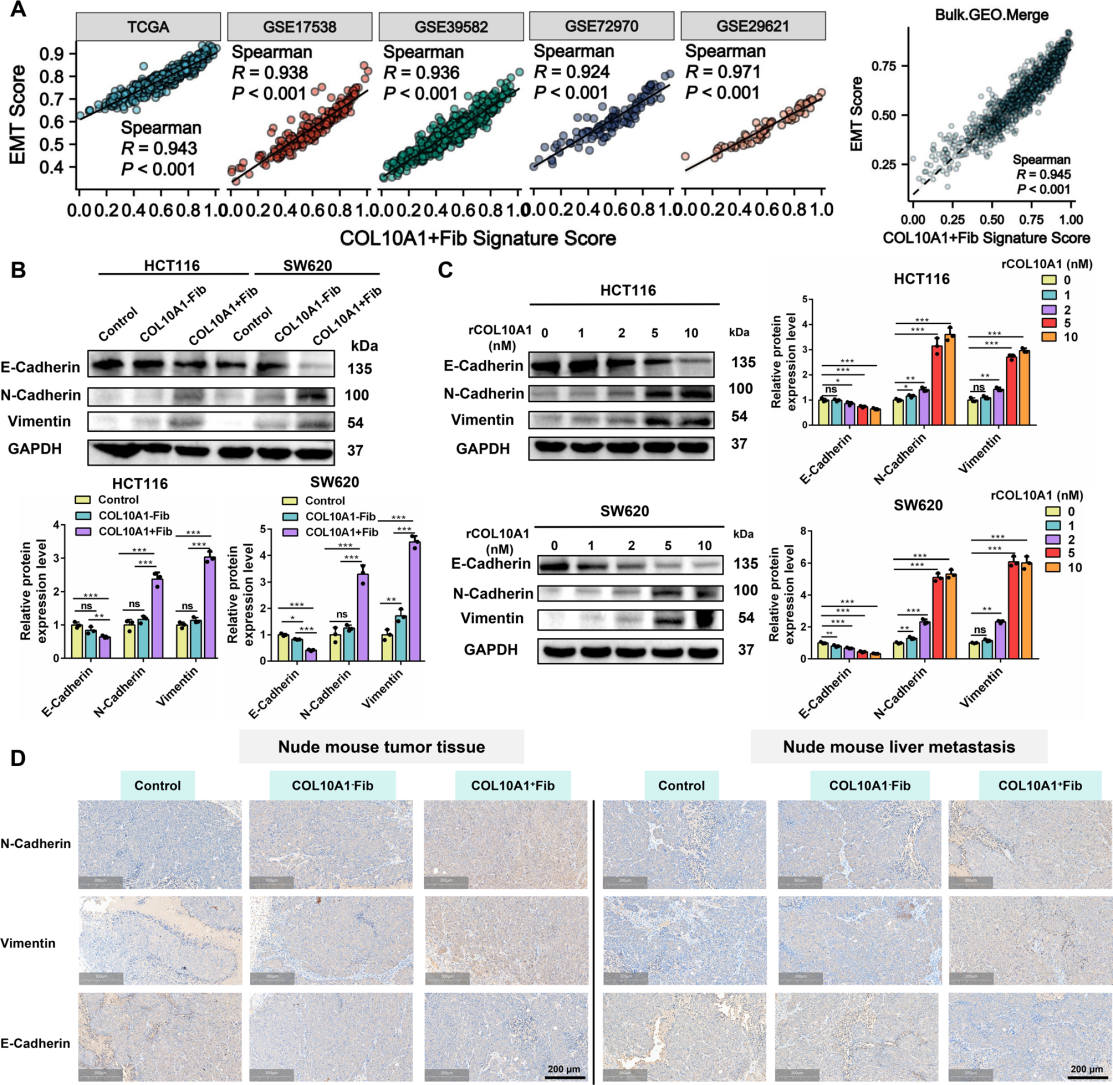
COL10A1^+^Fib promote epithelial–mesenchymal transition (EMT) in CRC cells. **A**. Spearman correlation analysis between COL10A1^+^Fib infiltration levels and EMT scores across multiple bulk datasets (TCGA, GSE17538, GSE39582, GSE72970, GSE29621, and Bulk.GEO.Merge). **B**. Western blot analysis and quantification of EMT markers (E-cadherin, N-cadherin, vimentin) in HCT116 and SW620 cells co-cultured with COL10A1^+^Fib. **C**. Dose-dependent induction of EMT markers in HCT116 and SW620 cells treated with recombinant human COL10A1 (rCOL10A1) at 0, 1, 2, 5, and 10 nM. **D**. IHC (per group: *n* = 6) analysis of EMT marker expression (E-cadherin, N-cadherin, vimentin) in subcutaneous xenografts and liver metastases in nude mice. Scale bars, 300 μm. >*P < 0.05; **P < 0.01; ***P < 0.001

### Functional characterization of COL10A1^+^Fib in promoting metastasis and immunosuppression

Functionally, GSVA showed that COL10A1^+^Fib were enriched in EMT, TGF-β, and other pro-metastatic and immunosuppressive pathways (Supplementary Fig. 5A). GSEA of overlapping differentially expressed genes further supported their involvement in metastasis and immune modulation (Supplementary Fig. 5B–C). In both the TCGA and Bulk.GEO.Merge datasets, high COL10A1^+^Fib infiltration was associated with enrichment of immunosuppressive and metastatic pathways (Supplementary Fig. 5D–E). In ten CRC liver metastasis single-cell datasets, COL10A1^+^Fib were enriched in metastatic lesions and exhibited consistent functional profiles (Supplementary Fig. 5F–H). Notably, COL10A1^+^Fib infiltration was highest in the CMS4 molecular subtype, which is known for strong metastatic potential and immune evasion [24] (Supplementary Fig. 5I). Together, these results suggest that COL10A1^+^Fib may contribute to pro-metastatic and immunosuppressive processes in the TME.

### Identification of cell subpopulations in spatial proximity to COL10A1^+^Fib

To further elucidate the interactions of COL10A1^+^Fib within the TME, we performed re-clustering of the six major non-fibroblast cell types in the Merge.ScRNA dataset. This analysis identified 10 epithelial subclusters, 7 myeloid subclusters, 9 T/NK cell subclusters, 6 endothelial subclusters, 7 plasma cell subclusters, and 6 B cell subclusters (Supplementary Figure. 6 A). Together with the previously defined 11 fibroblast subclusters, this yielded a total of 56 annotated cellular subpopulations. Next, we integrated spatial transcriptomic (ST) data from four CRC patients to map the spatial distribution of each cell subcluster (Supplementary Figure. 6B) and calculated their spatial distances to COL10A1^+^Fib. We focused particularly on immune and epithelial lineages. Among epithelial cells, Epi_1 was found to be the closest in spatial proximity to COL10A1^+^Fib. For myeloid populations, the nearest subclusters were APOE^+^Macr, SPP1^+^Macr, and C1QA^+^Macr (Supplementary Figure. 6 C).

### COL10A1 signaling network analysis

Cell–cell communication analysis, centered on the COL10A1 signaling axis, revealed that Epi_1 and the three aforementioned macrophage subclusters (APOE^+^Macr, SPP1^+^Macr, and C1QA^+^Macr) were the primary recipients of COL10A1 signaling (Supplementary Figure. 7 A–C). These findings suggest that COL10A1^+^Fib may influence the invasive potential of tumor epithelial cells and modulate the immunological behavior of macrophages through COL10A1-mediated signaling, potentially contributing to metastasis and immunosuppressive microenvironment formation.

### COL10A1^+^Fib promotes malignant progression of CRC cells

To investigate the role of COL10A1^+^Fib in promoting the malignant progression of CRC, we comprehensively assessed their effects on tumor cell phenotypes. Spatial transcriptomic data revealed that COL10A1^+^Fib is spatially adjacent to the epithelial subcluster Epi_1, which was also identified as a primary recipient of COL10A1 signaling. Epi_1 was significantly enriched in tumor tissues, with the highest proportion observed in late-stage TNM classifications (Supplementary Figure. 8 A). Functional enrichment analysis showed that Epi_1 strongly activated malignant signaling pathways, including EMT, TGF-β, and stemness (Supplementary Figure. 8B), and was associated with poor patient prognosis (Supplementary Figure. 8 C). CNV analysis further indicated that Epi_1 exhibited marked genomic instability (Supplementary Figure. 8D–E), supporting its malignant phenotype. The marker genes of Epi_1, ERO1A and ALDOA, were found to be spatially colocalized with COL10A1^+^Fib (Primary Tumor: *n* = 35) (Supplementary Figure. 8 F, Figure. 4 A), suggesting local interactions may enhance epithelial malignancy. In vitro, we generated stable COL10A1^+^Fib and COL10A1⁻Fib lines (Supplementary Figure. 9 A–B). Co-culture experiments (Figure. 4B)demonstrated that COL10A1^+^Fib significantly promoted HCT116 cell proliferation (Figure. 4 C) and colony formation (Figure. 4D), with similar results observed in the SW620 cell line (Supplementary Figure. 9 C–D). In vivo, subcutaneous xenograft models showed that COL10A1^+^Fib markedly accelerated tumor growth and Ki67 expression (per group: *n* = 6) (Figure. 4E–H). We additionally included a “normal CAF + HCT116” group: these CAFs enhanced tumor growth relative to the Ctrl and COL10A1⁻Fib groups, yet remained significantly less potent than COL10A1^+^Fib (per group: *n* = 6) (Supplementary Fig. 9E-G). Transwell assays confirmed that COL10A1^+^Fib enhanced CRC cell migration and invasion (Figure. 4I–J, Supplementary Figure. 9 H). In the splenic injection liver metastasis model, mice co-injected with COL10A1^+^Fib developed significantly more liver metastases (per group: *n* = 4) (Figure. 4 K), suggesting a strong pro-metastatic effect in vivo.

### COL10A1^+^Fib promote epithelial–mesenchymal transition (EMT) in CRC cells

Mechanistically, analysis across multiple CRC bulk transcriptomic datasets revealed that COL10A1^+^Fib infiltration was highly correlated with EMT scores (*R* > 0.9, *P* < 0.05) (Figure. 5 A, Supplementary Fig. 5C). Western blot analysis showed that co-culture with COL10A1^+^Fib reduced E-cadherin and increased N-cadherin and vimentin in HCT116 and SW620 cells (Figure. 5B), indicating EMT activation. Treatment with recombinant human COL10A1 protein (rCOL10A1) induced EMT markers in a dose-dependent manner (Figure. 5C) with consistent results validated by immunofluorescence (Supplementary Figure. 9I). Immunohistochemistry (IHC) of both subcutaneous and metastatic liver tissues confirmed that COL10A1^+^Fib significantly upregulated EMT markers in vivo (per group: *n* = 6) (Figure. 5D). In summary, COL10A1^+^Fib promotes EMT in CRC cells through COL10A1 secretion, and synergistically enhances proliferation, migration, and metastasis, supporting a contributory role in the tumor microenvironment of CRC.

### COL10A1^+^Fib promotes M2-like macrophage polarization

**Fig. 6:**
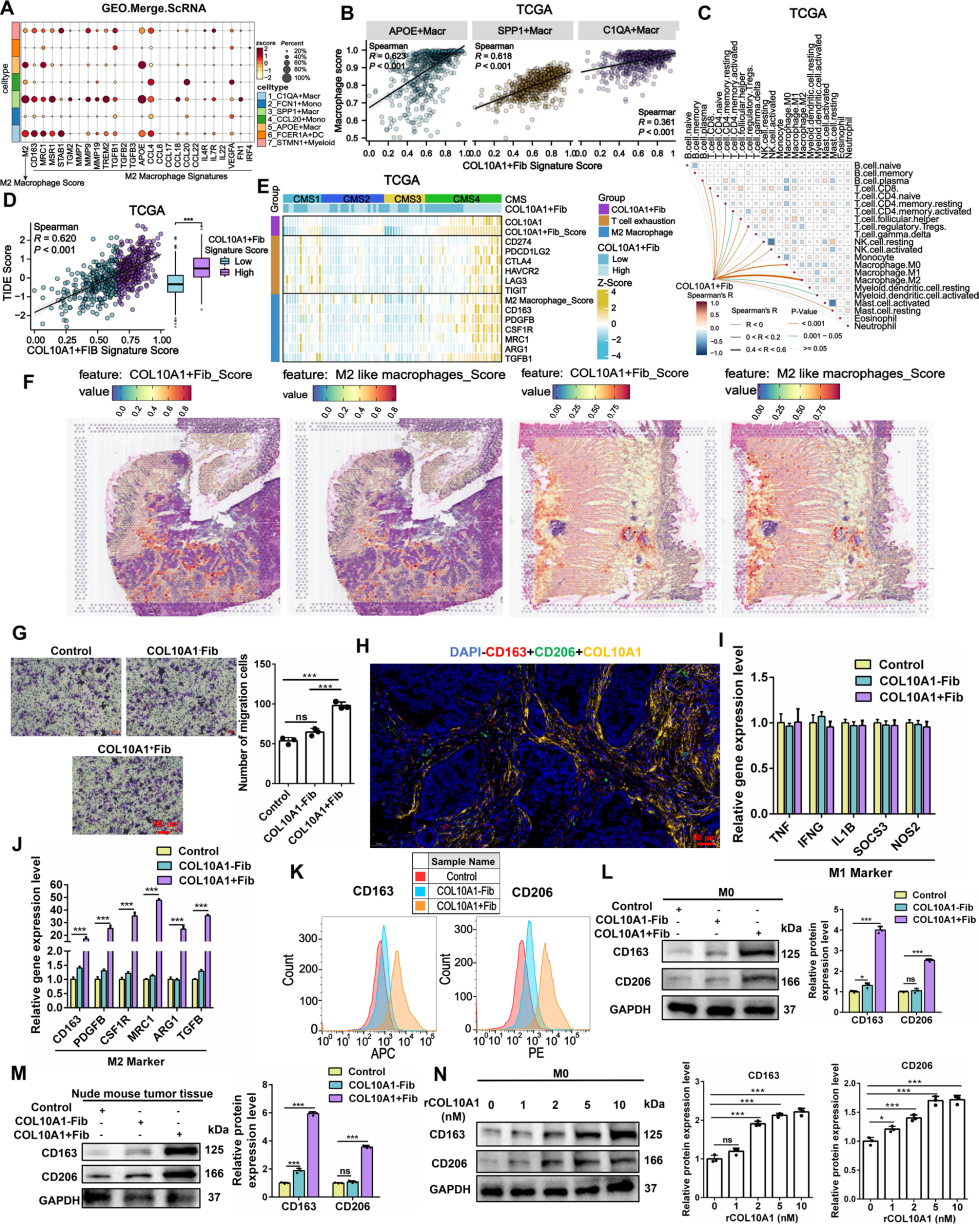
COL10A1^+^Fib promotes M2-like macrophage polarization. **A**. Heatmap showing M2 macrophage scores across myeloid cell subpopulations in the Merge.ScRNA dataset. **B**. Spearman correlation analysis between COL10A1^+^Fib infiltration and APOE^+^Macr, SPP1^+^Macr, and C1QA^+^Macr subpopulations in the TCGA dataset

Previous analyses revealed that M2-like macrophage subtypes (APOE^+^Macr, SPP1^+^Macr, and C1QA^+^Macr) are the primary recipients of COL10A1 signaling, exhibiting consistently high M2 scores (Fig. 6A). Across the seven principal cell lineages, APOE, SPP1, and C1QA are expressed almost exclusively within the myeloid compartment, and, within the myeloid subclusters, each gene is largely restricted to its cognate macrophage subset—APOE to APOE^+^Macr, SPP1 to SPP1^+^Macr and C1QA to C1QA^+^Macr (Supplementary Fig. 10A). In the TCGA cohort, COL10A1^+^Fib showed strong positive correlations with the infiltration of these M2 subpopulations (*R* > 0.3, *P* < 0.001) (Fig. 6B). Immune infiltration analysis using CIBERSORTx identified the strongest association between COL10A1^+^Fib and M2 macrophages (Fig. 6C), alongside elevated TIDE dysfunction and exclusion scores (Fig. 6D). Moreover, in the CMS4 molecular subtype, expression levels of COL10A1, COL10A1^+^Fib, T cell exhaustion markers, M2 markers, and M2 macrophage scores were all significantly increased (Fig. 6E), suggesting a close link between COL10A1^+^Fib and the immunosuppressive TME. Spatial transcriptomics further confirmed the colocalization of COL10A1^+^Fib with M2-like macrophages (Fig. 6F; Supplementary Fig. 10B). Functionally, COL10A1^+^Fib significantly promoted the migration of M0 macrophages (Fig. 6G) and localized near CD163^+^ and CD206^+^ M2 macrophages (Primary Tumor: *n* = 35) (Fig. 6H). qPCR, flow cytometry, and Western blot analysis demonstrated that COL10A1^+^Fib upregulated multiple M2 markers (CD163, CD206) without affecting M1 markers (Fig. 6I–L). In subcutaneous tumors in nude mice, the COL10A1^+^Fib group also exhibited pronounced M2 polarization characteristics (Fig. 6M). Recombinant COL10A1 protein (rCOL10A1) induced M2 marker expression in a dose-dependent manner (Fig. 6N), indicating that COL10A1 plays an important role in M2 polarization.

### COL10A1^+^Fib promotes M2-like polarization through the CD18/JAK1/STAT3 signaling axis

**Fig. 7:**
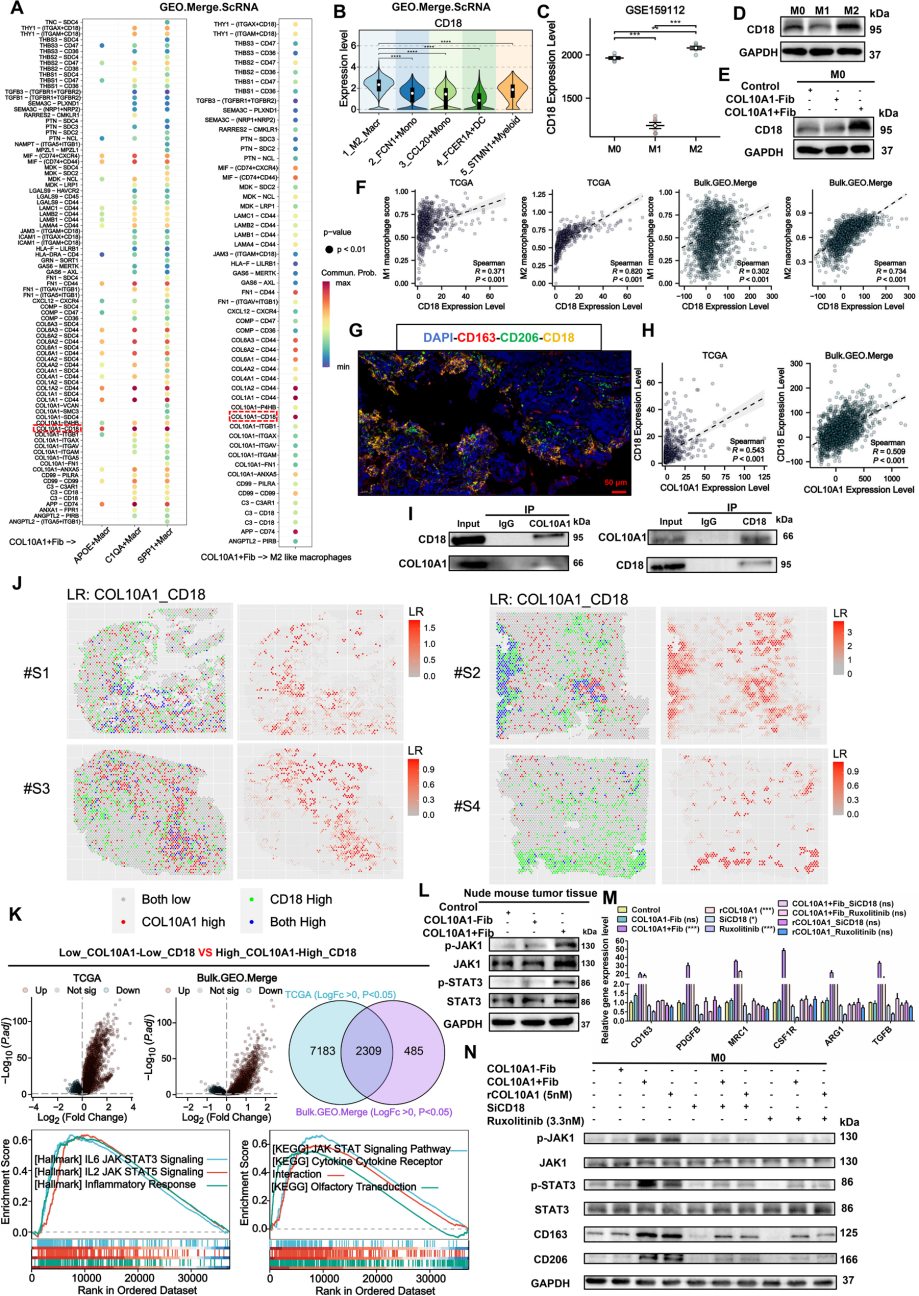
COL10A1^+^Fib promotes M2-like polarization through the CD18/JAK1/STAT3 signaling axis. **A**. Cell–cell communication analysis in the Merge.ScRNA dataset showing COL10A1^+^Fib interacting with APOE^+^Macr, SPP1^+^Macr, and C1QA^+^Macr via COL10A1 signaling. **B**. CD18 expression levels across myeloid subpopulations in single-cell data, highest in M2 macrophages. **C**. CD18 gene expression in M0, M1, and M2 macrophages in the GSE159112 dataset. **D**. Western blot showing CD18 protein levels under M0, M1, and M2 induction conditions. **E**. Western blot comparison of CD18 protein expression in M0 macrophages treated with COL10A1⁻Fib versus COL10A1^+^Fib. **F**. Spearman correlation analysis between CD18 infiltration and M1/M2 macrophage infiltration in TCGA and Bulk.GEO.Merge datasets. **G**. Multiplex immunofluorescence staining (DAPI, CD163^+^, CD206^+^, CD18^+^) showing CD18 co-localized with M2 markers (Primary Tumor: *n* = 35). Scale bars, 50 μm. **H**. Spearman correlation analysis of COL10A1 and CD18 expression in TCGA and Bulk.GEO.Merge cohorts. **I**. Co-immunoprecipitation (Co-IP) assay confirming direct interaction between COL10A1 and CD18 proteins. **J**. Spatial transcriptomic evidence of COL10A1 and CD18 colocalization in CRC tissue. **K**. GSEA enrichment based on TCGA and Bulk.GEO.Merge datasets showing activation of JAK/STAT3 signaling downstream of the COL10A1/CD18 axis. **L**. Western blot analysis of p-JAK1, p-STAT3, CD163, and CD206 in subcutaneous tumors of nude mice. **M**. RT-qPCR analysis of M2-associated genes (CD163, PDGFB, MRC1, CSF1R, ARG1, TGFB) under the indicated treatments: Control_CM, COL10A1^+^Fib-CM, COL10A1^−^Fib-CM, recombinant COL10A1 (rCOL10A1, 5 nM), siCD18, Ruxolitinib (3.3 nM), and their combinations. **N**. Western blot analysis of p-JAK1, p-STAT3, CD163, and CD206 in M0 macrophages subjected to the indicated treatments: Control_CM, COL10A1^+^Fib-CM, COL10A1^−^Fib-CM, recombinant COL10A1 (rCOL10A1, 5 nM), siCD18, Ruxolitinib (3.3 nM), and their combinations. **P < 0.01; ***P < 0.001; ****P < 0.0001

Mechanistically, cell–cell communication analysis implicated COL10A1-CD18 as a major candidate ligand–receptor pair mediating interactions between COL10A1^+^Fib and M2-like macrophages (Fig. 7A). CD18 expression was highest in M2 macrophages (Fig. 7B–D; Supplementary Fig. 11A) and further upregulated upon stimulation with COL10A1^+^Fib (Fig. 7E; Supplementary Fig. 11B). In both TCGA and Bulk.GEO.Merge datasets, CD18 infiltration levels correlated moderately with M1 macrophages (*R* = 0.3–0.4) and strongly with M2 macrophages (*R* = 0.7–0.9) (Fig. 7F), supporting its association with M2 polarization. Multiplex immunofluorescence revealed CD18 localization predominantly in CD163^+^ or CD206^+^ macrophages (Primary Tumor: *n* = 35) (Fig. 7G), with strong positive correlation between COL10A1 and CD18 expression (*R* > 0.5, *P* < 0.001) (Fig. 7H). Protein–protein docking (Supplementary Fig. 11C–D) and co-immunoprecipitation assays (Fig. 7I) confirmed direct binding between COL10A1 and CD18, supported by spatial co-localization (Fig. 7J). GSEA indicated that the COL10A1/CD18 axis may promote M2 polarization through JAK1/STAT3 activation (Fig. 7K). In vivo experiments demonstrated that stimulation with COL10A1^+^Fib elevated p-JAK1/p-STAT3 levels and M2 marker expression (Fig. 7L). Intervention experiments showed that either CD18 knockdown (siRNA_2), JAK1/STAT3 inhibition with Ruxolitinib, or their combination effectively blocked COL10A1- or COL10A1^+^Fib-induced M2 polarization (Fig. 7M–N; Supplementary Fig. 11E). In summary, COL10A1^+^Fib appears to promote M2-like macrophage polarization via COL10A1-CD18-JAK1/STAT3 signaling, potentially contributing to an immunosuppressive microenvironment in CRC.

### M2-like macrophages enhance COL10A1 expression in COL10A1^+^Fib via the TGF-β/RUNX2 signaling axis

**Fig. 8:**
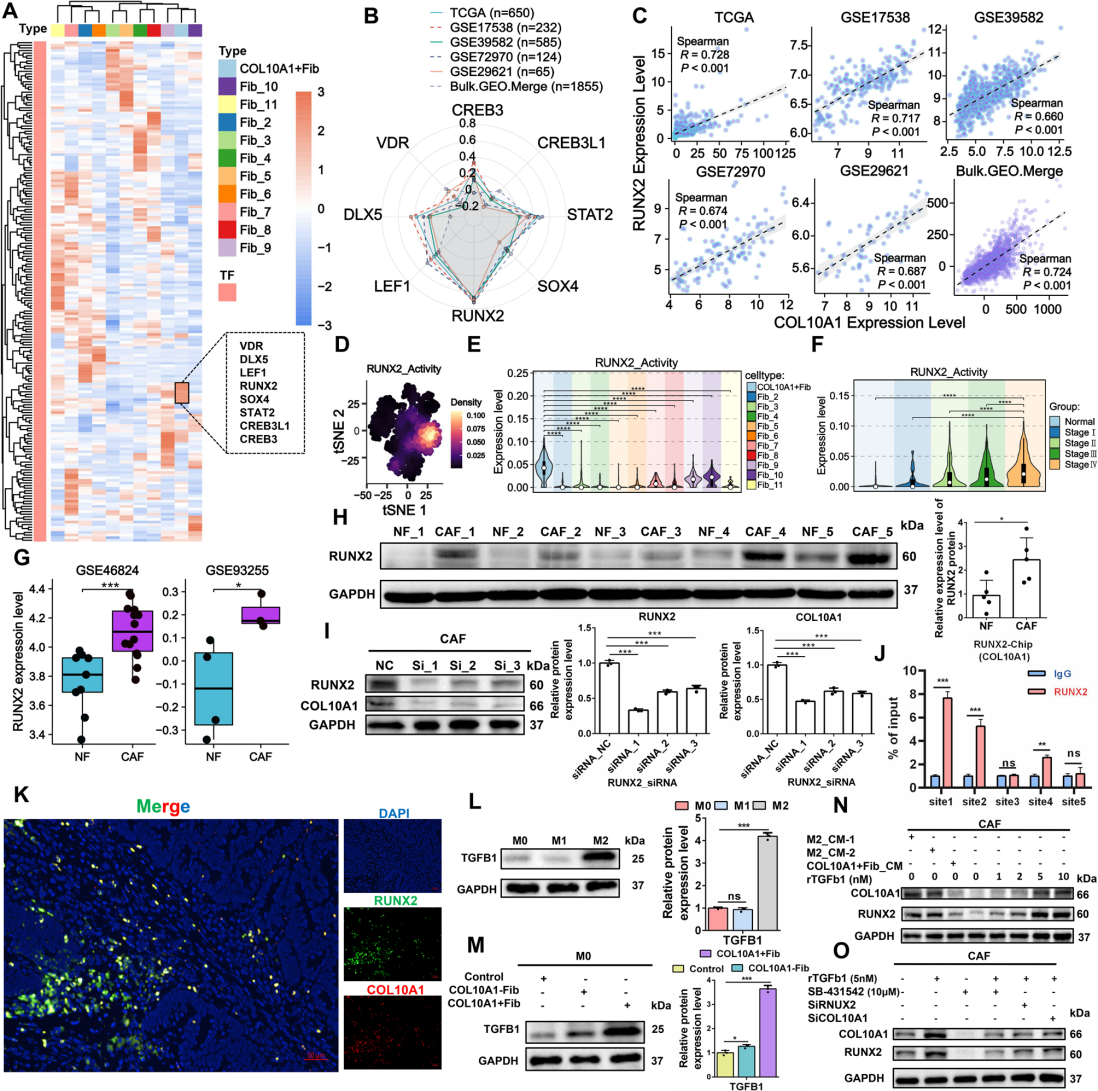
M2-like macrophages promote COL10A1 expression in COL10A1^+^Fib via the TGF-β/RUNX2 axis. **A**. Heatmap showing transcription factors activated in COL10A1^+^Fib based on SCENIC analysis. **B**. Radar plot of correlation coefficients between COL10A1 and candidate TFs (VDR, DLX5, LEF1, RUNX2, SOX4, STAT2, CREB3L1, CREB3) across bulk datasets (TCGA, GSE17538, GSE39582, GSE72970, GSE29621, Bulk.GEO.Merge). **C**. Scatterplots showing Spearman correlations between COL10A1 and RUNX2 in bulk datasets. **D**. t-SNE plot showing RUNX2 activity across cell types in single-cell RNA-seq. **E**. Violin plot of RUNX2 activity across fibroblast subpopulations. **F**. RUNX2 activity across TNM stages. **G**. RUNX2 expression in CAFs vs. NFs in GSE46824 and GSE93255 datasets. **H**. Western blot and quantification of RUNX2 protein in CAFs and NFs. **I**. Western blot showing downregulation of COL10A1 and RUNX2 after RUNX2 siRNA transfection. **J**. ChIP-qPCR results for RUNX2 binding at five predicted COL10A1 promoter sites. **K**. Multiplex immunofluorescence showing co-localization of RUNX2 and COL10A1 in fibroblasts (Primary Tumor: *n* = 35); Scale bars, 50 μm. **L**. Western blot and quantification of TGFB1 in M0, M1, and M2 macrophages. **M**. TGFB1 protein expression after M0 macrophages were induced by COL10A1^+^Fib. **N**. Western blot analysis of COL10A1 and RUNX2 expression in CAFs exposed to M2 macrophage-conditioned medium (M2_CM-1, M2_CM-2) or COL10A1^+^Fib conditioned medium (COL10A1^+^Fib_CM) or recombinant TGF-β1 (rTGF-β1) at 0, 1, 2, 5, and 10 nM. **O**. Western blot analysis of COL10A1 and RUNX2 protein levels under the indicated treatments: recombinant TGF-β1 (5 nM), SB-431,542 (10 µM), siRUNX2, siCOL10A1, and their combinations. *P < 0.05; ***P < 0.001

To elucidate upstream regulators of COL10A1 expression in COL10A1^+^Fib, we employed the SCENIC algorithm on the GEO.Merge.ScRNA dataset to identify potential transcription factors. The analysis revealed a set of transcription factor modules enriched in COL10A1^+^Fib, including VDR, DLX5, LEF1, RUNX2, SOX4, STAT2, CREB3L1, and CREB3 (Fig. 8A). Correlation analysis across multiple bulk RNA-seq datasets identified RUNX2 as the factor most strongly associated with COL10A1 expression (Spearman’s *R* > 0.6) (Figs. 8B–C), suggesting it may be a key transcriptional regulator. Single-cell data indicated that RUNX2 activity was concentrated in COL10A1^+^Fib and increased with CRC progression (Figs. 8D–F). RUNX2 was also significantly upregulated in CAFs compared to normal fibroblasts (Figs. 8G–H). Knockdown of RUNX2 resulted in marked reduction of COL10A1 expression (Fig. 8I), supporting a regulatory role. ChIP-qPCR confirmed that RUNX2 directly binds to Site1 and Site2 of the COL10A1 promoter (Fig. 8J), and multiplex immunofluorescence further showed co-localization of RUNX2 and COL10A1 in fibroblasts (Primary Tumor: *n* = 35) (Fig. 8K), indicating direct transcriptional activation. We next investigated how RUNX2 is regulated. M2-like macrophages emerged as the primary source of TGF-β signaling and robustly activated this pathway in COL10A1^+^Fib (Figs. 8L–M, Supplementary Fig. 12A–C). In vitro, the upregulation of RUNX2 and COL10A1 in CAFs treated with COL10A1^+^Fib-CM was observed; however, it was significantly lower than the upregulation of RUNX2 and COL10A1 in CAFs induced by M2 macrophage conditioned medium, either from IL-4 and IL-13 (M2_CM-1) or from COL10A1^+^Fib (M2_CM-2), and exogenous rTGF-β1 stimulation exhibited similar dose-dependent effects (Fig. 8N). Importantly, inhibition of TGF-β signaling with SB-431,542 or RUNX2 knockdown significantly reduced COL10A1 expression, which could be partially restored by rTGF-β1 addition (Fig. 8O). Collectively, these findings support the model that TGF-β secreted by M2-like macrophages induces RUNX2 activation, which in turn directly drives COL10A1 transcription, forming a TGF-β/RUNX2/COL10A1 positive feedback loop. This mechanism illustrates how immune cells promote the activation and maintenance of COL10A1^+^Fib, contributing to an immunosuppressive TME and disease progression.

### The small-molecule inhibitor NU7441 suppresses COL10A1 expression and secretion in COL10A1^+^Fib

**Fig. 9:**
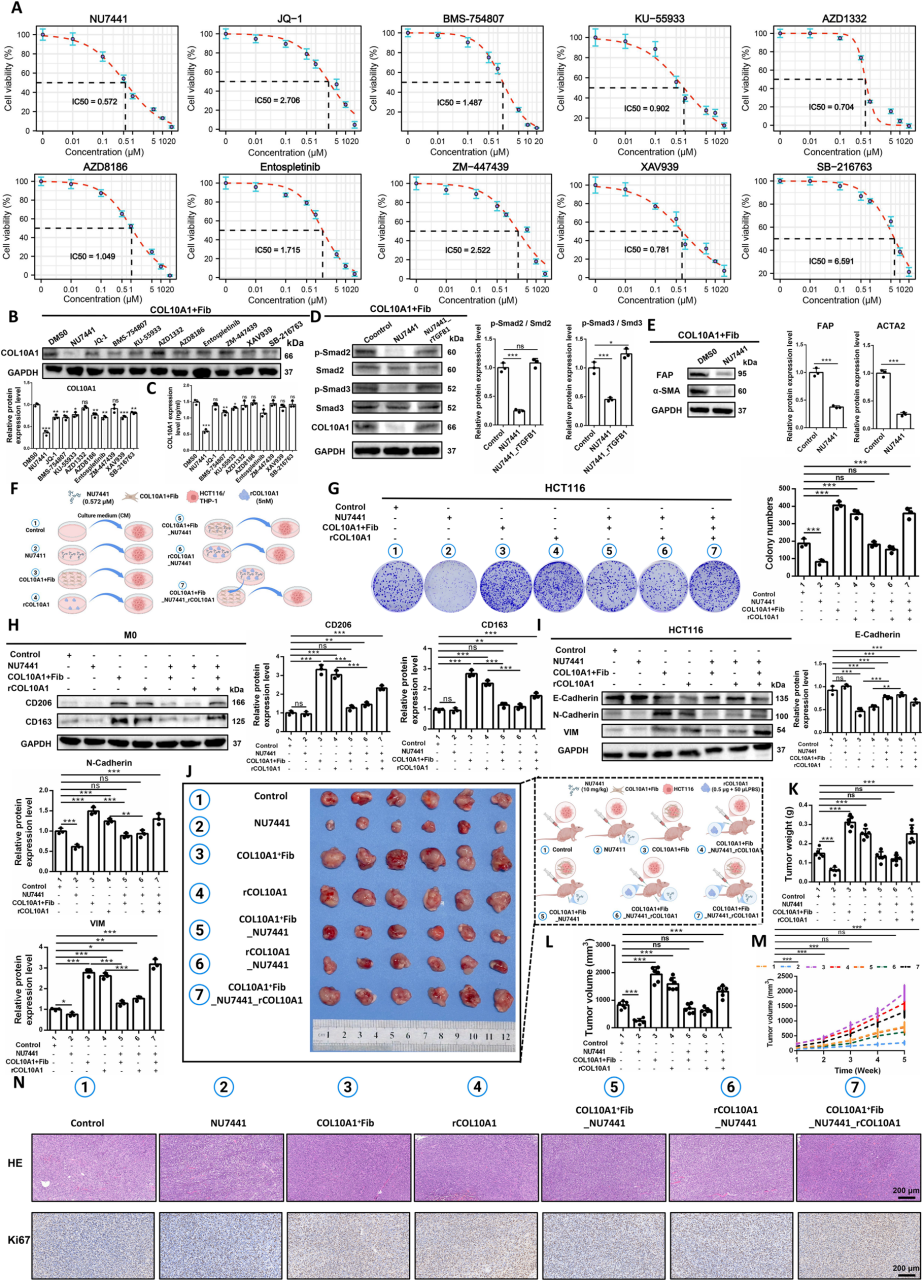
NU7441 suppresses COL10A1^+^Fib function and reverses its tumor-promoting effects on CRC cells in vitro and in vivo. **A**. IC50 curves of 10 small-molecule compounds against COL10A1^+^Fib cells. **B**. Western blot and densitometric quantification of COL10A1 expression in CAFs after treatment with the following compounds: NU7441 (0.572 µM), JQ-1 (2.706 µM), BMS-754,807 (1.487 µM), U-55,933 (0.902 µM), AZD1332 (0.704 µM), AZD8186 (1.049 µM), Entospletinib (1.715 µM), ZM-447,439 (2.522 µM), XAV939 (0.781 µM) and SB-216,763 (6.591 µM). **C**. ELISA quantification of COL10A1 secretion following treatment. **D**. Western blot demonstrating Smad2/3 pathway inhibition in CAFs treated with NU7441 (0.572 µM), recombinant TGF-β1 (rTGF-β1, 5 nM). **E**. Western blot analysis of FAP and α-SMA (ACTA2) expression in CAFs after treatment with NU7441 (0.572 µM). **F**. Schematic overview of the experimental design: conditioned media (CM) collected from CAFs subjected to recombinant COL10A1 (rCOL10A1, 5 nM), NU7441 (0.572 µM), or their combination were applied separately to HCT116 cells and M0 macrophages for downstream assays. **G**. Colony formation assay assessing proliferation of HCT116 under various treatments. **H**. Western blot and quantification of CD163 and CD206 expression in M0 macrophages across groups. **I**. Western blot and quantification of EMT markers (E-cadherin, N-cadherin, Vimentin) in HCT116. **J**. Representative images of xenograft tumors from each treatment group; rCOL10A1 (0.5 µg in 50 µL PBS), NU7441 (10 µg kg⁻¹). **K**. Final tumor weight comparisons across groups. **L**. Final tumor volume comparisons across groups. **M**. The line chart shows the tumor growth curves for seven experimental groups (per group: *n* = 6), including: Group 1 (Control), Group 2 (NU7441), Group 3 (COL10A1 + Fib), Group 4 (rCOL10A1), Group 5 (COL10A1 + Fib_NU7441), Group 6 (rCOL10A1_NU7441), and Group 7 (COL10A1 + Fib_NU7441_rCOL10A1). **N**. H&E and Ki67 immunohistochemistry staining of xenograft tumors (per group: *n* = 6). Scale bars, 200 μm. *P < 0.05; **P < 0.01; ***P < 0.001

To identify potential compounds capable of inhibiting the function of COL10A1^+^Fib, we utilized the oncoPredict algorithm to estimate the IC50 values of 198 drugs across the TCGA-CRC and Bulk.GEO.Merge cohorts. Ten candidate compounds were found to be significantly negatively correlated with COL10A1^+^Fib infiltration in both datasets (Supplementary Figs. 13 A–C), and their binding to COL10A1 protein was evaluated via molecular docking (Supplementary Fig. 13D). In vitro, NU7441 significantly reduced both the expression and secretion of COL10A1 in COL10A1^+^Fib (Figs. 9A–C). NU7441, as a DNA-PKcs inhibitor, has been extensively studied for its ability to enhance tumor sensitivity to radiation and chemotherapy. We observed that NU7441 significantly inhibited the phosphorylation of Smad2/3, but no significant changes were observed in TGFB1 levels (Fig. 9D, Supplementary Fig. 14A). NU7441 also downregulated the expression of COL10A1 and RUNX2 in COL10A1^+^Fib (Fig. 9D, Supplementary Fig. 14B), while reducing the expression of CAF markers FAP and α-SMA (Fig. 9E), indicating effective inhibition of COL10A1^+^Fib functional state. Co-culture experiments (Fig. 9F) further demonstrated that NU7441 significantly impaired the pro-tumorigenic effects of COL10A1^+^Fib-conditioned medium (CM) and recombinant COL10A1 protein (rCOL10A1), reducing CRC cell proliferation (Fig. 9G), M2 macrophage polarization (Fig. 9H), CD18/JAK1/STAT3 signaling pathway activation (Supplementary Fig. 14C), and EMT marker expression (Fig. 9I). Molecular dynamics simulations showed that NU7441 forms a stable complex with COL10A1, supported by favorable RMSD, hydrogen bond count, RMSF, radius of gyration (Rg), and SASA indices (Supplementary Figs. 14D–H), suggesting strong binding stability and functional inhibition. In vivo, NU7441 significantly suppressed tumor growth in HCT116 xenograft models. While COL10A1^+^Fib and rCOL10A1 individually promoted tumor growth, NU7441 treatment reversed their pro-tumorigenic effects. When both COL10A1^+^Fib and rCOL10A1 were administered together, NU7441 partially mitigated the combined effect (per group: *n* = 6) (Figs. 9J–N). Collectively, NU7441 reduces RUNX2 and COL10A1 by blocking Smad2/3 phosphorylation and simultaneously disrupts CD18/JAK1/STAT3 signalling, further underscoring its candidacy as a therapeutic agent targeting COL10A1^+^Fib.

### Widespread presence and conserved functional pattern of COL10A1^+^Fib subpopulation pan-cancers

**Fig. 10:**
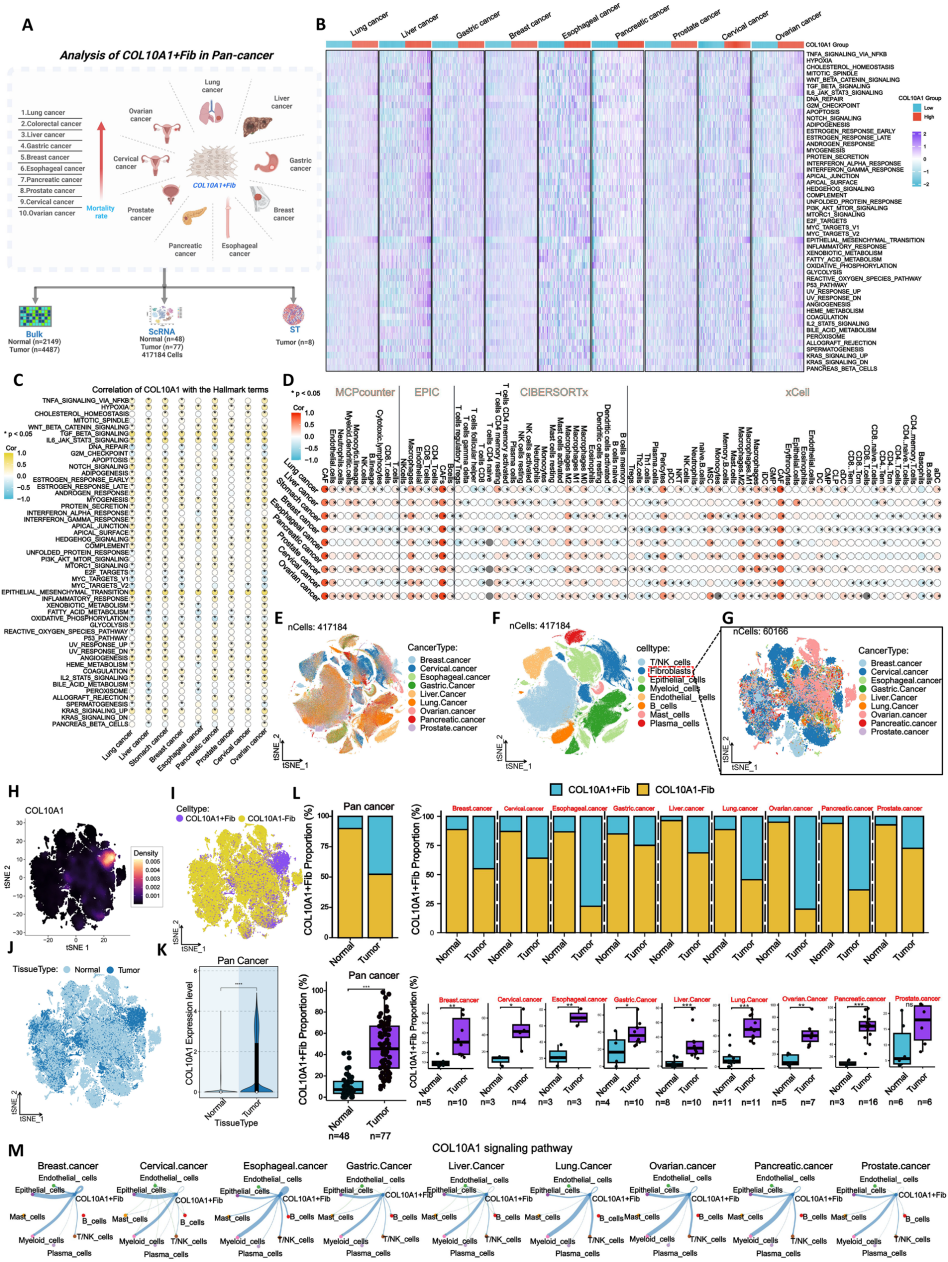
Distribution and Functional Characterization of COL10A1^+^Fib Subpopulations Pan-Cancers. **A**. Integrated analysis of bulk RNA-seq, ScRNA, and spatial transcriptomics (ST) data across nine solid tumors (lung, liver, gastric, breast, esophageal, pancreatic, prostate, cervical, and ovarian cancers) to systematically assess COL10A1^+^Fib characteristics. **B**. GSVA heatmap showing enrichment of tumor-promoting pathways in COL10A1-high expression groups across nine cancer types. **C**. Bubble plot of Spearman correlations between COL10A1 expression and hallmark pathways. **D**. Correlation bubble plot between COL10A1 expression and immune cell infiltration levels, assessed using four algorithms: MCPcounter, EPIC, CIBERSORTx, and xCell. **E**. t-SNE plot of 417,184 single cells from pan-cancer scRNA-seq data, colored by cancer type. **F**. t-SNE plot showing distribution of major cell types across all cancers. **G**. t-SNE plot of fibroblast subclusters, colored by cancer type. **H**. Density plot showing COL10A1 expression distribution within fibroblast populations. **I**. t-SNE plot showing spatial distribution of COL10A1^+^Fib versus COL10A1⁻Fib within fibroblasts. **J**. t-SNE comparison of fibroblast distributions in normal versus tumor tissues. **K**. Violin plots showing differences in COL10A1 gene expression between tumor and normal tissues across cancer types. **L**. (Top) Bar chart comparing the proportion of COL10A1^+^Fib in tumor versus normal tissues for each cancer type. (Bottom) Boxplot summarizing COL10A1^+^Fib abundance across cancers. **M**. Cell–cell communication network derived from scRNA-seq data showing active interactions between COL10A1^+^Fib and epithelial or myeloid cells via the COL10A1 signaling axis. *P < 0.05; **P < 0.01; ***P < 0.001

To systematically evaluate the distribution and functional characteristics of COL10A1^+^Fib across cancers, we integrated multi-omics data from ten high-mortality solid tumors (including CRC). This included bulk transcriptomic profiles (normal tissues *n* = 2,149; tumor tissues *n* = 4,487), single-cell transcriptomes (*n* = 417,184 cells), and spatial transcriptomics (*n* = 8 tumor samples; esophageal cancer data unavailable) (Fig. 10A). At the bulk level, COL10A1 was significantly upregulated in tumor tissues across all nine cancer types examined (Supplementary Fig. 15A) and its high expression was consistently associated with poorer OS, particularly in liver, gastric, breast, prostate, and cervical cancers (Supplementary Fig. 15B). GSVA and pathway correlation analyses revealed that COL10A1-high tumors exhibited enrichment in multiple pro-tumorigenic signaling pathways, including TNFA/NF-κB, TGF-β, WNT, IL6/JAK/STAT3, and EMT (Figs. 10B–C). Immune infiltration analysis using MCPcounter, EPIC, CIBERSORTx, and xCell demonstrated a strong positive correlation between COL10A1 expression and CAF or M2 macrophage abundance across all cancer types, while negative correlations were observed with CD8^+^ T cells and activated NK cells, although with variable significance across cancers (Fig. 10D). In single-cell RNA-seq datasets, eight major cell types were annotated (Figs. 10E–F, Supplementary Figs. 15 C–D). Fibroblasts were further subdivided into COL10A1^+^Fib and COL10A1⁻Fib based on COL10A1 expression (Figs. 10G–I). COL10A1^+^Fib was predominantly localized to tumor tissues, with its abundance and expression level significantly higher in tumors than in normal tissues across all cancers except prostate cancer (Figs. 10J–L, Supplementary Fig. 15E). Cell–cell communication analyses identified that COL10A1^+^Fib exhibited the most active interactions with epithelial and myeloid cells via the COL10A1 signaling axis (Fig. 10M). Spatial transcriptomic analysis revealed that COL10A1^+^Fib co-localized with regions enriched in EMT signatures, M2 macrophages, and high tumor cell density across all eight analyzed cancers (excluding esophageal cancer, for which ST data was not available) (Supplementary Fig. 16) These findings suggest a potential role for COL10A1^+^Fib in metastasis-related and immune-evasive processes. In summary, our multi-omics pan-cancer analysis reveals the widespread presence of COL10A1^+^Fib across solid tumors. This fibroblast subset shows similar transcriptomic features linked to immunosuppression and metastasis across multiple tumors, supporting its promise as a therapeutic target that warrants further validation.

## Discussion

CRC is a common and highly prevalent malignancy worldwide. Patients with advanced-stage disease frequently develop distant metastases and generally show limited responses to immunotherapy [25–27]. CAFs play a crucial role in CRC progression and immune evasion; however, the pathogenic subpopulations and underlying mechanisms remain poorly understood [28]. Through integrative multi-omics analysis, this study systematically identified a COL10A1^+^Fib that is enriched in late-stage CRC. This subpopulation was shown to significantly promote tumor metastasis and immunosuppression and is closely associated with poor prognosis. Pan-cancer analysis suggested that COL10A1^+^Fib is prevalent in multiple solid tumors and displays similar transcriptomic features. These findings highlight the pathogenic features of COL10A1^+^Fib and provide novel insights into TME-targeted therapies and cross-cancer treatment strategies.

Most current studies on COL10A1 have primarily focused on its expression and function in tumor cells [13], while investigations into its role within CAF subpopulations—particularly COL10A1^+^Fib—remain limited. Existing reports indicate that COL10A1 is highly expressed in the tumor stroma of breast, pancreatic, and gastrointestinal cancers, where its upregulation is typically associated with increased tumor invasiveness and poorer patient survival [29]. Notably, matrix-producing CAFs (matCAFs) characteristically express high levels of COL10A1, and knockdown of COL10A1 in breast and gastric cancer mouse models significantly suppresses tumor proliferation and metastasis [30]. In CRC, COL10A1 overexpression is linked to perineural invasion, lymph node metastasis, and higher histological grade [31]. ScRNA in basal cell carcinoma further identified COL10A1 as a CAF-specific gene, rarely expressed in other cell types, and enriched in highly invasive tumors [32]. In breast cancer, elevated COL10A1 expression correlates with reduced tumor-infiltrating lymphocytes (TILs) and diminished immune cell infiltration, suggesting a potential role in shaping an immunosuppressive microenvironment [29, 33]. Moreover, studies in CRC confirm that COL10A1 is more abundantly expressed in CAFs than in cancer cells [31]. However, prior research has largely described the phenotypic expression of COL10A1 in CAFs without systematically elucidating its cellular origin or functional mechanisms. In this study, we comprehensively identified a CRC-associated CAF subpopulation—COL10A1^+^Fib—and confirmed that CAFs are the principal source of COL10A1 in CRC. We further demonstrated that COL10A1^+^Fib exhibits both immunosuppressive and pro-metastatic functions, and for the first time, systematically dissected the underlying regulatory mechanisms using COL10A1^+^Fib as a defined cellular model.

Single-cell sequencing studies have confirmed the high heterogeneity of CAFs across a range of solid tumors, with distinct subpopulations driving tumor progression and immune regulation through secretion of factors or matrix remodeling [34, 35]. For instance, CD36^+^ CAFs in hepatocellular carcinoma can uptake oxidized low-density lipoprotein and activate the p38-CEBP-MIF axis to recruit MDSCs, promoting an immunosuppressive environment [36]. In CRC, IL1R1^+^ CAFs facilitate immune evasion through IL-1 signaling and are associated with T cell suppression, where targeting this pathway improves immunotherapeutic response [37]. FAP^+^ CAFs have also been shown to form an immune barrier with SPP1^+^ macrophages, limiting T cell infiltration [38]. Building on these findings, our study is the first to reveal the role of COL10A1^+^Fib as a central communication hub connecting tumor cells, immune cells, and the extracellular matrix. We demonstrate that COL10A1^+^Fib promotes epithelial-to-mesenchymal transition (EMT) in tumor epithelial cells via COL10A1 collagen secretion, significantly enhancing their migration and invasion. COL10A1^+^Fib promotes M2 macrophage polarization via the COL10A1/CD18/JAK1/STAT3 signaling axis, contributing to an immunosuppressive microenvironment. COL10A1 binds integrin CD18 on macrophage surfaces, activating JAK1/STAT3 signaling and upregulating M2 markers such as CD163 and CD206. CD18 (ITGB2), a β-chain integrin, is a known receptor for collagen proteins [39, 40], and the JAK1/STAT3 pathway is a well-established driver of M2 polarization [41]. Our findings suggest for the first time that CAF-derived collagen may directly influence immune cell function through exogenous signaling. Furthermore, we show that M2 macrophages reciprocally activate COL10A1^+^Fib through a TGF-β/RUNX2 axis, forming a positive feedback loop. TGF-β secreted by M2 macrophages significantly upregulates RUNX2 in neighboring fibroblasts, thereby enhancing COL10A1 transcription. RUNX2, a master regulator of osteogenic differentiation, has also been implicated in fibrosis and pathological fibroblast activation, as its deletion blocks the transition from normal to pathological fibroblasts [42, 43]. Our data suggest that RUNX2 not only regulates fibrotic genes but also serves an important mediator linking immune responses and tumor progression. Collectively, this study establishes a bidirectional signaling loop between CAFs, tumor cells, and immune cells, positioning COL10A1^+^Fib as a potential pivotal cell type in metastasis-related immune suppression—providing new insight into CAF heterogeneity.

Notably, our analysis shows that the increases in COL10A1 expression, COL10A1^+^ fibroblast (COL10A1^+^Fib) infiltration, T-cell-exhaustion markers, M2 signatures, and M2-macrophage scores are most pronounced in the CMS4 molecular subtype of CRC. CMS4 is characterised by robust TGF-β signalling, a desmoplastic stroma, and poor responsiveness to immunotherapy—hallmarks of an immunosuppressive TME [24]. Mechanistically, these converging signals are consistent with our data: TGF-β directly up-regulates COL10A1 transcription in fibroblasts via RUNX2, driving the expansion of the COL10A1^+^Fib pool; reciprocally, the COL10A1^+^Fib secretome reinforces immunosuppression by polarising M0 macrophages toward an M2 phenotype through the COL10A1–CD18/JAK1–STAT3 axis and by promoting T-cell dysfunction/exclusion. The resulting M2 macrophages secrete additional TGF-β, establishing a positive-feedback loop that sustains both the COL10A1^+^Fib population and the immunosuppressive milieu. Thus, the co-enrichment of COL10A1^+^Fib and immunosuppressive programmes in CMS4 is not merely correlative but reflects an inter-dependent network driven by TGF-β signalling. This framework explains the particularly aggressive clinical behaviour and immune-checkpoint-blockade resistance of CMS4 tumours, and highlights COL10A1^+^Fib as a tractable stromal target for overcoming the immune-refractory nature of this subtype.

COL10A1^+^Fib emerges as a promising therapeutic target. Through drug sensitivity prediction and experimental validation, we identified NU7441 as a small-molecule inhibitor reduced COL10A1 expression/secretion and attenuated CAF-mediated pro-tumor effects in preclinical models. NU7441 is a selective DNA-PKcs inhibitor that has been widely used to sensitize tumors to radiation and chemotherapy [44, 45]. Recent studies also suggest that NU7441 exerts immunomodulatory effects by reducing the suppressive function of myeloid-derived suppressor cells (MDSCs) and enhancing T cell responses in immunotherapy-resistant tumors [46, 47]. Our study shows that NU7441 suppresses COL10A1 expression in CAFs primarily through inhibition of the TGF-β/Smad signaling pathway, consistent with prior findings in renal fibrosis models [48]. Notably, molecular dynamics simulations revealed stable binding between NU7441 and COL10A1 protein, providing theoretical support for direct molecular targeting. In vitro and in vivo experiments confirmed that NU7441 effectively blocks COL10A1^+^Fib-induced tumor proliferation, migration, EMT, and immune suppression. These findings suggest that NU7441 not only holds anti-tumor potential but also presents a drug repurposing opportunity for targeting pathological CAF subpopulations.

Through multi-omics integration, this study demonstrated that COL10A1^+^Fib are broadly enriched across nine prevalent solid tumor types, including lung, liver, gastric, and breast cancers. Remarkably, these cells show similar transcriptomic signatures linked to metastasis and immunosuppression across several tumor types. These findings align with the pan-cancer single-cell atlas presented by Luo et al., which identified a conserved CAF subpopulation characterized by endothelial–mesenchymal transition and spatial proximity to SPP1^+^ macrophages [49]. Notably, the molecular and spatial properties of COL10A1^+^Fib strongly resemble this “pan-cancer” CAF state, suggesting that COL10A1^+^Fib supporting the possibility that COL10A1^+^Fib constitutes a recurrent CAF subset in diverse tumor contexts. This expands our understanding of CAF heterogeneity and provides a unified framework to explain metastatic potential and immune evasion across multiple malignancies.

Despite the breadth of our multi-omics analyses, several caveats remain. First, all spatial transcriptomic data were cross-sectional, preventing us from charting the temporal emergence and functional plasticity of COL10A1^+^Fib during therapy or metastasis. Second, although we showed that COL10A1 engages CD18 to activate JAK1/STAT3 and induce M2 polarization, CD18 is a hub integrin that feeds into multiple pathways; the full downstream network, the structural basis of COL10A1–CD18 binding, and context-specific effects across immune subsets are still undefined and warrant high-resolution interactome and conditional-knockout studies. Finally, NU7441 reduced COL10A1 expression and curbed COL10A1^+^Fib activity, yet, as a DNA-PKcs inhibitor with pleiotropic targets, it lacks COL10A1 specificity, and its anti-CAF actions may extend beyond the observed TGF-β/Smad inhibition; future work must clarify these mechanisms, develop more selective COL10A1 inhibitors, and establish pharmacodynamic windows and safety profiles before clinical translation.

## Conclusion

Our study reveals a CAF subpopulation, COL10A1^+^Fib, associated with CRC progression and immune suppression, and suggests that this subpopulation may play a similar role in multiple major solid tumors. This finding warrants further exploration as a therapeutic target for CRC and other malignant tumors.

## Supplementary Information

Below is the link to the electronic supplementary material.

Supplementary Material 1

Supplementary Material 2

Supplementary Material 3

Supplementary Material 4

## Data Availability

The datasets supporting the conclusions of this article are included within the article and its additional files.

## Abbreviations

ARG1: Arginase 1
Bulk RNA-seq: Bulk RNA sequencing
CAF: Cancer-associated fibroblast
CD18: Cluster of differentiation 18
ChIP-qPCR: Chromatin immunoprecipitation followed by qPCR
COL10A1: Collagen type X alpha 1 chain
Co-IP: Co-immunoprecipitation
CMS: Consensus molecular subtype
CNV: Copy number variation
CRC: Colorectal cancer
ELISA: Enzyme-linked immunosorbent assay
EMT: Epithelial-mesenchymal transition
GEO: Gene expression omnibus
GSEA: Gene set enrichment analysis
GSVA: Gene set variation analysis
GTEx: Genotype-tissue expression
hdWGCNA: high‑dimensional weighted gene co‑expression network analysis
IF: Immunofluorescence
IHC: Immunohistochemistry
JAK1: Janus kinase 1
MDSC: Myeloid-derived suppressor cell
MRC1: Mannose receptor C type 1
PCA: Principal component analysis
qPCR: Quantitative polymerase chain reaction
RUNX2: Runt-related transcription factor 2
ssGSEA: Single-sample GSEA
scRNA-seq: Single-cell RNA sequencing
STAT3: Signal transducer and activator of transcription 3
ST: Spatial transcriptomics
TGF-β: Transforming growth factor-β
TCGA: The cancer genome atlas
TME: Tumor microenvironment
tSNE: t-distributed stochastic neighbor embedding
UMAP: Uniform manifold approximation and projection
WB: Western blot
