# Supplementary material for: COL10A1+ fibroblasts promote colorectal cancer metastasis and M2 macrophage polarization with pan-cancer relevance
Source: J Exp Clin Cancer Res. 2025 Aug 18;44:243. doi: 10.1186/s13046-025-03510-8 (PMC12360028; doi:10.1186/s13046-025-03510-8)
Supplement: Supplementary file 1 — Supplementary Material 1 [file 13046_2025_3510_MOESM1_ESM.docx]

**Supplementary Materials and Methods**

**Cell–Cell Communication and Developmental Trajectory Analysis**

Cell–cell communication networks were inferred using the “CellChat” R package[1], referencing both the built-in CellChatDB and external ligand–receptor interaction databases to elucidate intercellular signaling patterns[2]. Single-cell pseudotime trajectory analysis was conducted using the “Monocle 2” R package to reconstruct the dynamic transcriptional progression of different cellular states[3].

**High-Dimensional Weighted Gene Co-Expression Network Analysis (hdWGCNA)**

Weighted gene co-expression network analysis based on single-cell data was performed using the “hdWGCNA” R package. A scale-free gene network was constructed at the single-cell level. The ssGSEA algorithm was applied to score the activity of co-expression modules across fibroblast subpopulations. Hub genes within each module were identified and visualized using the HubGeneNetworkPlot function, with the top 25 hub genes selected for each module[4].

**Single-Cell Transcription Factor Analysis**

Single-cell transcriptional regulatory network analysis was performed using the SCENIC (Single-Cell Regulatory Network Inference and Clustering) framework in R, which infers transcription factor (TF) regulons and quantifies their activity at the single-cell level[5]. The analysis involved three key steps: (1) the GENIE3 algorithm was used to construct co-expression networks between TFs and candidate target genes; (2) motif enrichment analysis was conducted using the RcisTarget database to identify core regulatory targets with high confidence; and (3) the AUCell algorithm was applied to score the activity of each regulon in individual cells. These results were then integrated with cell subtype annotations and functional states to identify key TFs that may drive specific cell fate transitions and functional programs.

**Functional Enrichment Analysis**

Gene function annotation and pathway enrichment analysis were carried out using the “clusterProfiler” R package for Gene Ontology (GO) and Kyoto Encyclopedia of Genes and Genomes (KEGG) analysis. The Benjamini–Hochberg method was used to adjust for multiple hypothesis testing. Gene set enrichment analysis (GSEA) was also performed using “clusterProfiler”. Sample-level gene set activity was quantified with the “ssGSEA” algorithm from the “GSVA” R package, allowing estimation of biological process activity across different samples. HALLMARK gene sets were retrieved from the Molecular Signatures Database (MSigDB, https://www.gsea-msigdb.org/gsea/msigdb). Pathway activity inference for fibroblast subpopulations was conducted using the “decoupleR” R package[6].

**Immune Correlation Analysis**

To systematically assess the infiltration characteristics of immune and stromal cells within the TME, this study applied four mainstream algorithms—CIBERSORTx, xCell, EPIC, and MCPcounter—on bulk transcriptomic data across pan-cancer cohorts. CIBERSORTx analysis was performed via its official online platform (https://cibersortx.stanford.edu/) to estimate the relative abundance of 22 immune cell types[7]. The remaining algorithms were implemented using R packages: “xCell”[8] was used to infer scores for various immune and non-immune cell types; “MCPcounter”[9]was applied to quantify the absolute abundance of multiple immune and stromal cell subsets; and “EPIC”[10] estimated the relative composition of immune cells—including T cells, B cells, and macrophages—as well as cancer cells. The TIDE (Tumor Immune Dysfunction and Exclusion) algorithm was used to evaluate immune evasion in CRC samples by assessing T cell dysfunction in tumors with high cytotoxic T lymphocyte (CTL) infiltration and T cell exclusion in tumors with low CTL infiltration, thereby predicting immune suppression and potential response to immunotherapy[11].

**Prognostic Analysis**

Survival analysis, including overall survival (OS) and relapse-free survival (RFS), was conducted using the “survival” and “survminer” R packages. Kaplan–Meier survival curves were plotted to evaluate the impact of specific gene expression levels or cell subpopulation proportions on patient prognosis, with statistical significance assessed using Cox proportional hazards regression.

**Cell Culture and Polarization**

Multiple human colorectal cancer (CRC) cell lines, including HCT8, HCT15, HT29, HCT116, Caco-2, SW480, SW620, DLD1, LOVO, and RKO, were obtained from the Cell Bank of the Chinese Academy of Sciences. HCT116, RKO, and Caco-2 were cultured in high-glucose Dulbecco’s Modified Eagle Medium (DMEM; Gibco), supplemented with 10% fetal bovine serum (FBS) and 1% penicillin–streptomycin. SW480 and SW620 were maintained in Leibovitz’s L-15 medium (L15; Gibco) with 10% FBS and 1% penicillin–streptomycin. LOVO cells were cultured in F-12K medium (Kaighn’s Modification of Ham’s F-12 Medium; Gibco) supplemented with 10% FBS and 1% penicillin–streptomycin. HCT8, HCT15, HT29, and DLD1 were grown in RPMI-1640 medium (Gibco) with 10% FBS and 1% penicillin–streptomycin. All cells were maintained at 37°C in a humidified atmosphere containing 5% CO₂, except for SW480 and SW620, which were cultured in a CO₂-free. The human monocytic leukemia cell line THP-1 was used to establish an in vitro macrophage polarization model. THP-1 cells were cultured in complete RPMI-1640 medium containing 10% FBS and 1% penicillin–streptomycin and treated with 100 ng/mL phorbol 12-myristate 13-acetate (PMA; MCE, Cat. HY-18739) for 24 hours to induce differentiation into unpolarized M0 macrophages. To generate M1 macrophages, M0 cells were stimulated with 20 ng/mL interferon-γ (IFN-γ; MCE, Cat. HY-P7025) and 100 ng/mL lipopolysaccharide (LPS; MCE, Cat. HY-D1056A1) for 48 hours. For M2 polarization, M0 cells were treated with 20 ng/mL interleukin-4 (IL-4; MCE, Cat. HY-P70445) and 20 ng/mL interleukin-13 (IL-13; MCE, Cat. HY-P70568) for 48 hours. All commercially obtained cell lines were authenticated immediately after thawing by short tandem repeat (STR) profiling at the Cell Bank of the Chinese Academy of Sciences, and the resulting signatures were cross-checked against ATCC/DSMZ reference databases to rule out cross-contamination; authentication was repeated every six months. Mycoplasma contamination was assessed monthly with the MycoAlert™ PLUS Mycoplasma Detection Kit (Lonza, Cat. LT07-710); all cultures remained mycoplasma-negative for the entire duration of the study.

**Isolation and Culture of CAFs and Normal Fibroblasts (NFs)**

CAFs and NFs used in this study were obtained from our in-house biobank, and their isolation and characterization were performed as described in our previous publication[12]. Briefly, fresh CRC tumor tissues and paired adjacent normal tissues were minced and digested at 37°C for 2 hours with agitation in a digestion buffer containing 1 mg/mL collagenase (Sigma-Aldrich, Cat. C4-BIOC), DMEM (KeyGen, Cat. KGM12800), and 10% fetal bovine serum (FBS). The digested mixture was centrifuged, and the pellet was filtered through a 100 μm cell strainer. The resulting cells were resuspended and plated in DMEM supplemented with 10% FBS. After 2 hours of incubation, strongly adherent fibroblasts were selectively enriched.Fibroblasts were identified based on the expression of canonical markers. Immunofluorescence and Western blot analyses confirmed that the isolated cells expressed α-smooth muscle actin (α-SMA) and vimentin, but not epithelial marker KRT20 or myogenic marker desmin, indicating a high-purity CAF or NF population. These cells were subsequently used in co-culture assays, secretome analyses, and functional validations.

**Generation of Stable COL10A1⁺ and COL10A1⁻ Fibroblast Lines**

To generate fibroblast subpopulations with stable overexpression or knockout of COL10A1, a lentiviral system was employed. COL10A1⁺Fib were established by infecting CAFs with a lentiviral vector encoding human COL10A1 cDNA (Syngentech, Beijing), followed by puromycin selection. Successful overexpression was confirmed by fluorescence microscopy and Western blotting. For COL10A1⁻ fibroblasts (COL10A1⁻Fib), CRISPR-Cas9-mediated gene knockout was performed using a lentiviral vector containing COL10A1-specific single-guide RNAs (sgRNAs) driven by a U6 promoter. Three sgRNA sequences were designed and screened for efficiency: sgRNA1 (TCTTGTTAGTGCCAACCAGG), sgRNA2 (GATGGTCCTAAGGGTAACCC), and sgRNA3 (AAACCAGGCTACGGAAGTCC). Following puromycin selection, Western blotting was used to validate COL10A1 knockdown and identify successfully edited clones. All stable cell lines underwent multiple passages to confirm stable growth and viability before being used in downstream functional and co-culture experiments. The lentiviral infection process is as follows: Lentiviral particles were produced with a second-generation packaging system (psPAX2 + pMD2.G; Addgene #12260/#12259) by triple-plasmid transfection of HEK-293T cells using polyethyleneimine (PEI, 1 mg mL⁻¹); culture supernatants were harvested at 48 h and 72 h, passed through 0.45 µm filters, concentrated with 100 kDa ultrafiltration columns. Primary CAFs (2 × 10⁵ cells well⁻¹, six-well plates) were transduced at an MOI of 30 in medium containing polybrene (8 µg mL⁻¹) using a 60-min spin-infection at 800 g, followed by 12 h incubation at 37 °C before medium replacement. Twenty-four hours later, puromycin (2 µg mL⁻¹) selection was applied for 7 days (empty-vector controls treated identically).

**Total Protein Extraction and Western Blot Analysis**

Total protein extraction and Western blot (WB) analyses were performed following standard protocols. For cellular samples, cells were washed twice with ice-cold PBS and lysed in RIPA buffer (Beyotime) supplemented with protease and phosphatase inhibitors for 15 minutes. Lysates were homogenized by pipetting and incubated on ice for an additional 30 minutes. For tissue samples, fresh frozen tissues were ground in liquid nitrogen, lysed in RIPA buffer, and centrifuged at 12,000 rpm for 15 minutes at 4°C. The supernatant was collected as total protein extract. Protein concentrations were quantified using a BCA assay kit (Beyotime). Equal amounts of protein (20–40 µg) were mixed with 5× SDS loading buffer, denatured at 100°C for 5 minutes, separated via SDS-PAGE (10% or 12% gels), and transferred onto PVDF membranes (Millipore). Membranes were blocked in 5% non-fat milk or BSA at room temperature for 2 hours and then incubated with primary antibodies overnight at 4°C. Primary antibodies used included: COL10A1 (ab58632, Abcam), E-Cadherin (A20798, Abclonal), N-Cadherin (A5286, Abclonal), Vimentin (A19607, Abclonal), CD163 (68218-1-Ig, Proteintech), CD206 (18704-1-AP, Proteintech), CD18 (ab52920, Abcam), JAK1 (310108, Zenbio), Phospho-JAK1 (310040, Zenbio), STAT3 (R22785, Zenbio), Phospho-STAT3 (310019, Zenbio), RUNX2 (ab236639, Abcam), TGF-β1 (A2124, Abclonal), Smad2 (A0440, Abclonal), Phospho-Smad2 (AP1342, Abclonal), Smad3 (A16913, Abclonal), Phospho-Smad3 (AP1263, Abclonal), ACTA2 (A1011, Abclonal), FAP (A6349, Abclonal), and GAPDH (60004-1-Ig, Proteintech) as internal control. After washing, membranes were incubated with HRP-conjugated secondary antibodies—either goat anti-rabbit IgG (SA00001-2, Proteintech) or goat anti-mouse IgG (SA00001-1, Proteintech)—for 2 hours at room temperature. Protein bands were visualized using enhanced chemiluminescence (ECL, Thermo Fisher) and imaged with a Bio-Rad chemiluminescence imaging system. Band intensities were quantified using ImageJ software and normalized to GAPDH expression.

**Immunofluorescence (IF) Staining**

Cell immunofluorescence: Cells were seeded onto coverslips in 24-well plates and grown to 60–70% confluence. Cells were fixed with 4% paraformaldehyde for 15 minutes, permeabilized with 0.2% Triton X-100 for 10 minutes, and washed three times with PBS. After blocking in 5% bovine serum for 1 hour, primary antibodies were added and incubated overnight at 4°C. After PBS washes, fluorescent secondary antibodies (Goat Anti-Mouse or Goat Anti-Rabbit Alexa Fluor 488/594, Invitrogen) were applied for 1 hour at room temperature in the dark. Nuclei were counterstained with DAPI (Beyotime) for 5 minutes. Slides were mounted and imaged using a Zeiss LSM880 confocal microscope.

Tissue immunofluorescence: Paraffin-embedded sections (4 μm) were dewaxed in xylene, rehydrated through graded ethanol, and subjected to antigen retrieval in 10 mM citrate buffer (pH 6.0) by microwave heating for 10–15 minutes. Sections were fixed in 4% paraformaldehyde for 5 minutes and blocked in 5% bovine serum for 1 hour. The remaining procedures were identical to cell IF, including primary antibody incubation, fluorescent secondary antibody staining, DAPI counterstaining, and imaging. PBS was used as a negative control in place of primary antibodies to assess non-specific staining.

Antibodies used included: COL10A1 (sc-59954, Santa Cruz), ACTA2 (A1011, Abclonal), ALDOA (67453-1-Ig, Proteintech), ERO1A (67416-1-Ig, Proteintech), RUNX2 (A2851, Abclonal), CD163 (68218-1-Ig, Proteintech), CD206 (18704-1-AP, Proteintech), CD18 (ab52920, Abcam), and FAP (A6349, Abclonal).

**In Vitro Treatment with Recombinant Proteins and Pathway Inhibitors**

To investigate the regulatory mechanisms of specific signaling pathways in cellular function, this study utilized recombinant proteins and small-molecule inhibitors in in vitro experiments. Recombinant human COL10A1 protein (rCOL10A1, Abnova, Cat# H00001300-P01) and recombinant human TGF-β1 protein (rTGFB1, MCE, HEK293-expressed) were applied to cell cultures at concentrations recommended by the manufacturer for 48 hours to simulate exogenous stimulation. For pathway inhibition, JAK1 inhibitor Ruxolitinib (MCE, Cat# HY-50856) and TGF-β pathway inhibitor SB-431542 (MCE, Cat# SB-431542) were used. Working concentrations were determined based on each drug’s IC50 as specified in the manufacturer’s instructions, and all inhibitors were filtered for sterility prior to use.

**Transient Transfection Assays**

To assess the functional roles of target genes, multiple siRNAs targeting RUNX2, COL10A1, and CD18 were designed and synthesized (three siRNAs each for RUNX2 and COL10A1; four for CD18) by RiboBio Co., Ltd. (Guangzhou, China). Detailed sequences are listed in **Supplementary Table 5.** Transfections were performed using Lipofectamine™ 3000 (Invitrogen) according to the manufacturer’s protocol. Briefly, cells were seeded in 6-well or 24-well plates and transfected at 50–70% confluency using serum-free, antibiotic-free medium. siRNA-reagent complexes were incubated at room temperature for 15 minutes before being added to the culture. After 6 hours, the medium was replaced with fresh complete medium, and cells were harvested after 48–72 hours for RNA/protein extraction and downstream assays. Transfection efficiency was validated by Western blot, and only siRNAs that achieved >70% knockdown efficiency were used for functional studies. A non-targeting control siRNA (NC-siRNA) was included to exclude non-specific effects.

**RNA Extraction and RT-qPCR**

Total RNA was extracted from cells using RNA-easy Isolation Reagent (Vazyme, R701-01), followed by reverse transcription and quantitative PCR (RT-qPCR).

RNA extraction: After experimental treatment, culture medium was removed and cells were gently washed with PBS to eliminate residual medium. For cells cultured in 6-well plates, 500 μL of RNA-easy Isolation Reagent was added per well to fully lyse the cells, which were then transferred into RNase-free 1.5 mL EP tubes. Subsequently, 200 μL of RNase-free ddH₂O was added, mixed thoroughly, and incubated at room temperature for 5 minutes. The lysates were centrifuged at 12,000 g for 15 minutes, and the aqueous phase was carefully transferred to a new tube. An equal volume of isopropanol was added to precipitate RNA, followed by two washes with 75% ethanol prepared in RNase-free water. The RNA pellet was air-dried and dissolved in 20 μL RNase-free ddH₂O. RNA concentration and purity were assessed using a NanoDrop spectrophotometer (Thermo Fisher), and samples with OD260/OD280 ratios between 1.8 and 2.0 were considered acceptable.

cDNA synthesis: For reverse transcription, 1 μg of total RNA was used with the HiScript® III RT SuperMix for qPCR (+gDNA wiper) (Vazyme, R323-01). Briefly, 4 μL of 4× gDNA wiper Mix was added to the RNA and brought to a total volume of 16 μL with RNase-free ddH₂O, then incubated at 42°C for 2 minutes to remove genomic DNA. Subsequently, 4 μL of 5× HiScript III qRT SuperMix was added, and the mixture was incubated at 37°C for 15 minutes followed by 85°C for 5 seconds to complete the reverse transcription. cDNA was stored at –80°C until use.

RT-qPCR: Quantitative PCR was performed using the ChamQ Universal SYBR qPCR Master Mix (Vazyme, Q711-02) in a 20 μL reaction containing 10 μL 2× SYBR Mix, 0.4 μL forward primer (10 μM), 0.4 μL reverse primer (10 μM), 2 μL cDNA, and 7.2 μL RNase-free ddH₂O. The cycling conditions were as follows: initial denaturation at 95°C for 30 s, followed by 40 cycles of 95°C for 10 s and 60°C for 15 s. A melt curve analysis was performed at the end of the run. Each sample was analyzed in technical triplicates. Relative gene expression levels were calculated using the 2^–ΔΔCT method with GAPDH as the internal control. The primer sequences are listed in **Supplementary Table 6.**

**Cell Function Assays**

Cell Proliferation (CCK-8 Assay): Cell proliferation was assessed using a Cell Counting Kit-8 (CCK-8, Dojindo, Japan). Treated cells were seeded in 96-well plates at 2,000 cells/well in five replicates. At 24, 48, 72, and 96 hours, 10 μL of CCK-8 solution was added per well and incubated for 1–2 hours. Absorbance at 450 nm was measured using a microplate reader (BioTek), and growth curves were generated from OD values.

Colony Formation Assay: For clonogenic analysis, cells were seeded at low density (1,000 cells/well) in 6-well plates and cultured in complete medium for 10–14 days. Medium was refreshed every 2–3 days. Once colonies (>0.5 mm diameter) became visible, cells were fixed with 4% paraformaldehyde for 20 minutes and stained with 0.5% crystal violet for 15 minutes. After washing and drying, colonies were imaged under a light microscope and manually counted. Each experiment was performed in triplicate.

Cell Migration and Invasion Assays (Transwell): Cell motility was evaluated using Transwell inserts (8.0 μm pores, Corning). For migration assays, cells were resuspended in serum-free medium and seeded in the upper chamber (5×10⁴–1×10⁵ cells/well), while medium with 10% FBS was added to the lower chamber as a chemoattractant. After 24 hours, non-migrated cells were removed, and migrated cells were fixed in 4% paraformaldehyde and stained with 0.5% crystal violet. Five random fields were photographed and counted per well. For invasion assays, Matrigel (Corning) was pre-coated in the upper chamber and allowed to solidify at 37°C prior to seeding. The rest of the procedure was consistent with the migration assay. The average number of penetrated cells in five fields per group was used for statistical analysis.

All functional experiments were independently repeated at least three times. Results are presented as mean ± standard deviation (SD).

**Flow Cytometry Analysis**

To evaluate the expression of surface markers in macrophage subpopulations, flow cytometry was performed for phenotypic analysis. After treatment, cells were harvested and washed twice with PBS, followed by blocking with 1% bovine serum albumin (BSA) to reduce non-specific binding. Cell suspensions were then incubated with fluorescently labeled monoclonal antibodies for 30 minutes at 4°C in the dark. The antibodies used included anti-CD163 (clone eBioGHI/61, APC-conjugated, eBioscience™, Thermo Fisher, Cat# 17-1639-42) and anti-CD206 (clone 19.2, PE-conjugated, eBioscience™, Thermo Fisher, Cat# 12-2069-42). After staining, cells were washed twice with PBS and resuspended for acquisition using a flow cytometer (BD FACSCanto II). Cells were analysed on a BD FACSCanto II flow cytometer using the following workflow: debris were excluded by gating on FSC‑A versus SSC‑A, and singlets were isolated with FSC‑H versus FSC‑W. Within this gate, fluorescence was acquired in two channels—APC for CD163‑APC (excitation 651 nm, emission 660 nm) and PE for CD206‑PE (excitation 565 nm, emission 576 nm). A minimum of 10,000 events per sample were collected for downstream analysis in FlowJo software (Tree Star, USA).

**Chromatin Immunoprecipitation Followed by qPCR (ChIP-qPCR)**

To verify whether the transcription factor RUNX2 directly binds to the COL10A1 promoter region, potential RUNX2-binding sites were predicted using the JASPAR database (https://jaspar.elixir.no/). The top five high-scoring sites were selected for ChIP-qPCR validation. Cells were fixed with 1% formaldehyde for 15 minutes at room temperature to cross-link DNA-protein complexes. The reaction was quenched with 0.125 M glycine, and cells were washed twice with cold PBS before lysis. Cells were then lysed in 990 μL ChIP Sonication Buffer with 10 μL protease inhibitors (100×) and subjected to sonication on ice (30% amplitude, 30 seconds on/30 seconds off, 5 cycles), yielding chromatin fragments of 200–500 bp. Ten microliters of lysate were used to assess fragment size and concentration. The remaining chromatin was diluted with High Salt and Lysis Buffer to 500 μL with added protease inhibitors. A 25 μL aliquot was reserved as 5% input and stored at −20°C. The rest was incubated overnight at 4°C with 5 μg anti-RUNX2 antibody. The next day, rProtein A/G MagPoly magnetic beads were added and incubated for 1 hour at 4°C. Beads were sequentially washed twice with High Salt and Lysis Buffer, once with Low Salt Buffer, and once with TE Buffer. The immunoprecipitated complexes were eluted with 150 μL ChIP buffer containing 2 μL Proteinase K and 2 μL RNase, then incubated at 65°C for 4 hours for reverse cross-linking. The purified DNA was analyzed by qPCR using the following primer pairs for the five predicted binding sites:

Site1: F: TTATTACCTAACATATAGCCTTGTA; R: GTATAGTGTAAGAGTAAACACTT

Site2: F: GACTCTGTGCTGAATACAGTCT; R: GGGAGTTGACTAGGCCAAGTG

Site3: F: AGCTCAGCCATACAGGCATAA; R: GTGGAGCCTAAGCTGACTGTAAG

Site4: F: GGACTAGAGATGCCAGGATGC; R: GCTCTTATATAACTTGTAAAGGT

Site5: F: CCTCCTTTGAAAGGAGGATTCCT; R: GCACAGAGTCAGGCAGTTTG

**Protein–Protein Docking and Co-immunoprecipitation (Co-IP)**

To predict the interaction between COL10A1 and CD18, the HDOCK online platform (http://hdock.phys.hust.edu.cn/) was employed for protein–protein docking analysis[13]. HDOCK integrates template-based modeling and free docking to identify potential binding sites. Amino acid sequences or 3D structures of COL10A1 and CD18 were retrieved from the AlphaFold Protein Structure Database[14]. Default parameters were used during the docking process, and the top 10 binding models were visualized. To validate protein interaction in cells, co-immunoprecipitation (Co-IP) assays were performed. Cells were lysed in NP-40 buffer supplemented with protease inhibitors, and lysates were clarified by centrifugation at 12,000 rpm for 15 minutes at 4°C. Equal amounts of total protein (500–800 μg) were incubated overnight at 4°C with 5 μg of anti-COL10A1 or anti-CD18 antibody. The next day, Protein A/G magnetic beads (Thermo Fisher) were added and incubated for an additional 1–2 hours at 4°C. Beads were then washed three times with cold PBS and eluted in SDS loading buffer, followed by denaturation at 95°C for 5 minutes. The samples were separated by SDS-PAGE and analyzed by Western blotting using antibodies against COL10A1 and CD18 to confirm co-precipitation, thereby validating protein–protein interaction.

**ELISA Assay**

A human COL10A1 ELISA kit (Human Collagen alpha-1(X) chain, COL10A1 ELISA Kit; Abclonal, RK08178) was used to quantify COL10A1 levels. Culture supernatants were collected and centrifuged to remove debris before analysis. All reagents and samples were equilibrated to room temperature for 30 minutes prior to the assay. Procedures were conducted according to the manufacturer's protocol. Briefly, reconstituted standards were serially diluted in standard diluent (R1) to concentrations ranging from 0–40 ng/mL and added to antibody-coated microplate wells along with the samples (100 μL per well), followed by incubation at 37°C for 2 hours. Biotin-labeled antibody, Streptavidin-HRP, and TMB substrate were then sequentially added, with incubations at 37°C and appropriate washing between steps. After reaction termination, absorbance was measured at 450 nm using a microplate reader. Each sample and standard was measured in duplicate. Final concentrations were calculated based on a four-parameter logistic regression fitted standard curve.

**Screening of Candidate Compounds Targeting COL10A1⁺Fib**

To identify potential small‑molecule inhibitors of COL10A1⁺ fibroblasts (COL10A1⁺ Fib), we used the oncoPredict R package. A regression model was first trained on transcriptome‑based IC50 data from the Genomics of Drug Sensitivity in Cancer (GDSC) cell‑line panel (https://gdsc‑combinations.depmap.sanger.ac.uk/). The resulting weights were applied to the normalised expression matrices of the TCGA‑CRC and Bulk.GEO.Merge cohorts to predict IC50 values for 198 small molecules. For each cohort, Spearman correlations were calculated between the COL10A1⁺ Fib signature score and the predicted IC50 of every drug; compounds showing a significant negative correlation in both cohorts were retained, yielding ten candidate agents. The top candidates included: NU7441 (MCE, HY-11006), JQ1 (MCE, HY-78695), BMS-754807 (MCE, HY-10200), KU-55933 (MCE, HY-12016), AZD1332 (MCE, HY-11006), AZD8186 (Alomone Labs, A-495), Entospletinib (MCE, HY-15968), ZM447439 (MCE, HY-10128), XAV939 (MCE, HY-15147), and SB216763 (MCE, HY-12012). These ten compounds were tested in vitro at gradient concentrations in COL10A1⁺Fib cells to determine their respective IC50 values. Following 24-hour treatment at IC50 concentrations, total protein was extracted and COL10A1 expression was assessed via Western blot. COL10A1 secretion in cell supernatants was quantified using ELISA to evaluate the inhibitory effect of each compound on protein secretion. Molecular dynamics (MD) simulations were conducted using GROMACS 2020.6 with the AMBER99SB-ILDN force field. Protein-ligand docking complexes were obtained from the Protein-Ligand Interaction Profiler (PLIP) and solvated in a TIP3P water box with appropriate Na⁺/Cl⁻ ion neutralization. Energy minimization was performed using the steepest descent algorithm, followed by temperature equilibration to 300 K and 100 ps of NVT and NPT equilibration steps. Production MD was run for 100 ns with data recorded every 10 ps. Long-range electrostatics were handled using the Particle Mesh Ewald method, with a 10 Å cutoff for non-bonded interactions. Stability and dynamic behavior of the complexes were evaluated using RMSD, RMSF, number of hydrogen bonds, radius of gyration (Rg), and solvent-accessible surface area (SASA). Data analysis was performed in GROMACS, with visualization using GraphPad Prism and PyMOL.

**Splenic Injection Liver Metastasis Model in Nude Mice**

All animal procedures were approved by the Institutional Animal Care and Use Committee of Nanjing First Hospital. Six-week-old BALB/c nude mice (both sexes) were obtained from Zhejiang Vital River Laboratory Animal Technology Co., Ltd., and maintained under specific pathogen-free (SPF) conditions. To assess the role of COL10A1⁺Fib in promoting CRC liver metastasis, a splenic injection model was established. Human CRC cell line HCT116 was stably transduced with luciferase and selected using 2 μg/mL puromycin. HCT116 cells were mixed with either COL10A1⁺Fib or COL10A1⁻ fibroblasts (COL10A1⁻Fib) at a 5:1 ratio and adjusted to a final concentration of 2.5×10⁴ cells/μL. A total of 1.0×10⁶ cells in 40 μL volume was injected per mouse. Mice were anesthetized with isoflurane, and following antiseptic preparation with povidone-iodine, the spleen was exposed via laparotomy. Cell suspension was injected into the splenic parenchyma using a sterile syringe. The abdominal wall and skin were sutured, and mice were monitored daily for general condition, body weight, and wound healing. On day 35 post-injection, mice were anesthetized and intraperitoneally injected with D-luciferin (15 mg/mL in sterile DPBS) at a dose of 150 mg/kg. After 10 minutes, bioluminescence imaging was performed using the IVIS Spectrum system to assess hepatic metastasis. Livers were harvested post-imaging, and visible metastatic nodules on the surface were manually counted to quantify metastatic burden.

**Subcutaneous Xenograft Model in Nude Mice**

To evaluate the functional role of COL10A1⁺Fib in tumor growth and test the in vivo efficacy of the COL10A1 inhibitor NU7441 and recombinant COL10A1 protein (rCOL10A1), a subcutaneous xenograft model was established using six-week-old BALB/c nude mice (both sexes). HCT116 cells were mixed with either COL10A1⁺Fib or COL10A1⁻Fib at a 5:1 ratio, adjusted to 2.5×10⁴ cells/μL. Each mouse received 1.0×10⁶ total cells in a 40 μL injection, administered subcutaneously into the right dorsal flank under sterile conditions. For control groups, HCT116 cells alone were injected at the same dosage. Mice were monitored daily for behavior, skin integrity, and tumor development.

NU7441 Treatment: Based on in vitro IC50 (0.572 μM), NU7441 (MCE, HY-11006) was administered intraperitoneally at 10 mg/kg, diluted in sterile saline. The treatment schedule consisted of 5 consecutive days of dosing followed by 2 days off, continued until the study endpoint. NU7441 administration began once palpable tumors were established. Mice were weighed regularly to monitor drug tolerability and potential toxicity.

Intratumoral rCOL10A1 Injection: Given that 5 nM rCOL10A1 significantly promoted CRC cell migration and invasion in vitro, the estimated effective in vivo dose was 0.5 μg per injection. To enhance local bioavailability and reduce systemic degradation, rCOL10A1 was administered intratumorally. Specifically, 0.5 μg of rCOL10A1 dissolved in 50 μL of sterile PBS was injected along the tumor margin every 3 days using a fine-gauge syringe.

Tumor dimensions (length, L; width, W) were measured every 2–3 days using electronic calipers, and tumor volume was calculated using the formula V = (L × W²)/2. Upon experimental completion, mice were euthanized, and tumors were excised, weighed, and subjected to histological and molecular analyses.

**Hematoxylin and Eosin (H&E) Staining**

To assess histopathological changes in tumor and liver metastatic tissues, standard hematoxylin and eosin (H&E) staining was performed. Tissue specimens were fixed in 10% neutral-buffered formalin for 24–48 hours, followed by graded ethanol dehydration, xylene clearing, and paraffin embedding. Sections were cut at 4 μm thickness and incubated at 60°C for 1 hour. After dewaxing and rehydration, sections were stained with hematoxylin for 5–8 minutes, washed in running water, differentiated in 1% acid alcohol for several seconds, and blued in alkaline water. Subsequently, sections were counterstained with 0.5% eosin for 1–3 minutes and rinsed with distilled water. After dehydration and clearing, slides were mounted using neutral resin. Stained sections were examined under a light microscope to evaluate morphological features such as tissue architecture, tumor cell density, and necrotic areas.

**Immunohistochemistry (IHC)**

Paraffin-embedded sections (4 μm) underwent standard deparaffinization, rehydration, and antigen retrieval. Endogenous peroxidase activity was blocked with 3% hydrogen peroxide for 10 minutes at room temperature. After blocking with 5% bovine serum for 30 minutes, sections were incubated overnight at 4°C with primary antibody against Ki67 (Proteintech, 27309-1-AP). The following day, slides were washed with PBS and incubated with an HRP-conjugated secondary antibody for 1 hour. Signal detection was performed using a DAB substrate kit (ZSGB-BIO), followed by hematoxylin counterstaining, dehydration, mounting, and microscopic imaging for analysis.

**References**

1. Jin S, Guerrero-Juarez CF, Zhang L, Chang I, Ramos R, Kuan CH, Myung P, Plikus MV, Nie Q. Inference and analysis of cell-cell communication using CellChat. Nature communications. 2021;12(1):1088.

2. Kirouac DC, Ito C, Csaszar E, Roch A, Yu M, Sykes EA, Bader GD, Zandstra PW. Dynamic interaction networks in a hierarchically organized tissue. Molecular systems biology. 2010;6:417.

3. Qiu X, Mao Q, Tang Y, Wang L, Chawla R, Pliner HA, Trapnell C. Reversed graph embedding resolves complex single-cell trajectories. Nature methods. 2017;14(10):979-82.

4. Morabito S, Reese F, Rahimzadeh N, Miyoshi E, Swarup V. hdWGCNA identifies co-expression networks in high-dimensional transcriptomics data. Cell reports methods. 2023;3(6):100498.

5. Aibar S, González-Blas CB, Moerman T, Huynh-Thu VA, Imrichova H, Hulselmans G, Rambow F, Marine JC, Geurts P, Aerts J, van den Oord J, Atak ZK, Wouters J, Aerts S. SCENIC: single-cell regulatory network inference and clustering. Nature methods. 2017;14(11):1083-6.

6. Badia IMP, Vélez Santiago J, Braunger J, Geiss C, Dimitrov D, Müller-Dott S, Taus P, Dugourd A, Holland CH, Ramirez Flores RO, Saez-Rodriguez J. decoupleR: ensemble of computational methods to infer biological activities from omics data. Bioinformatics advances. 2022;2(1):vbac016.

7. Rusk N. Expanded CIBERSORTx. Nature methods. 2019;16(7):577.

8. Aran D, Hu Z, Butte AJ. xCell: digitally portraying the tissue cellular heterogeneity landscape. Genome biology. 2017;18(1):220.

9. Yang S, Hu Y, Wang X, Deng M, Ma J, Hao Y, Ran Z, Luo T, Han G, Xiang X, Liu J, Shi H, Tan Y. Machine learning and deep learning to identifying subarachnoid haemorrhage macrophage-associated biomarkers by bulk and single-cell sequencing. Journal of cellular and molecular medicine. 2024;28(9):e18296.

10. Racle J, de Jonge K, Baumgaertner P, Speiser DE, Gfeller D. Simultaneous enumeration of cancer and immune cell types from bulk tumor gene expression data. eLife. 2017;6.

11. Jiang P, Gu S, Pan D, Fu J, Sahu A, Hu X, Li Z, Traugh N, Bu X, Li B, Liu J, Freeman GJ, Brown MA, Wucherpfennig KW, Liu XS. Signatures of T cell dysfunction and exclusion predict cancer immunotherapy response. Nature medicine. 2018;24(10):1550-8.

12. Liu X, Qin J, Nie J, Gao R, Hu S, Sun H, Wang S, Pan Y. ANGPTL2+cancer-associated fibroblasts and SPP1+macrophages are metastasis accelerators of colorectal cancer. Frontiers in immunology. 2023;14:1185208.

13. Yan Y, Tao H, He J, Huang SY. The HDOCK server for integrated protein-protein docking. Nature protocols. 2020;15(5):1829-52.

14. Jumper J, Evans R, Pritzel A, Green T, Figurnov M, Ronneberger O, Tunyasuvunakool K, Bates R, Žídek A, Potapenko A, Bridgland A, Meyer C, Kohl SAA, Ballard AJ, Cowie A, Romera-Paredes B, Nikolov S, Jain R, Adler J, Back T, Petersen S, Reiman D, Clancy E, Zielinski M, Steinegger M, Pacholska M, Berghammer T, Bodenstein S, Silver D, Vinyals O, Senior AW, Kavukcuoglu K, Kohli P, Hassabis D. Highly accurate protein structure prediction with AlphaFold. Nature. 2021;596(7873):583-9.
