# Supplementary material for: COL10A1+ fibroblasts promote colorectal cancer metastasis and M2 macrophage polarization with pan-cancer relevance
Source: J Exp Clin Cancer Res. 2025 Aug 18;44:243. doi: 10.1186/s13046-025-03510-8 (PMC12360028; doi:10.1186/s13046-025-03510-8)
Supplement: Supplementary file 2 — Supplementary Material 2 [file 13046_2025_3510_MOESM2_ESM.docx]

**Supplemental Figure**

**Supplementary Figure 1. Validation of Pro-Metastatic and Immunosuppressive Characteristics of Fibroblasts in Advanced CRC Based on ScRNA.GSE178341.**

A. UMAP plots of tissue type and TNM staging in the ScRNA.GSE178341 dataset.

B. Heatmap of surface marker gene expression across TNM stages.

C. UMAP distribution of the seven major cell types.

D. Circular plot of intercellular communication weights.

E. GSVA heatmap for fibroblast-specific functional enrichment across TNM stages.

**Supplementary Figure 2. COL10A1⁺Fib Selection and hdWGCNA Results.**

A. Violin plots showing expression of COL1A1, COL1A2, and COL3A1 across subclusters.

B. Heatmap of top three marker genes per fibroblast subcluster.

C–E. Soft threshold selection and module identification in hdWGCNA.

F–G. Bubble plot and tSNE showing enrichment of 16 gene modules across fibroblast subclusters.

H. Visualization of top 25 hub genes in the M12 module via co-expression network.

**Supplementary Figure 3. Validation of COL10A1⁺Fib Enrichment in the ScRNA.GSE178341 Cohort.**

A. tSNE map of COL10A1⁺Fib in ScRNA.GSE178341.

B. tSNE maps showing distribution of fibroblast subclusters by tissue type and TNM stage.

C. Pathway enrichment comparison between COL10A1⁺Fib and COL10A1⁻Fib.

D. Proportion of Fib_1 cells across TNM stages.

E. COL10A1 expression across TNM stages.

F. Pseudotime expression dynamics of COL10A1.

G. Proportional changes in COL10A1⁺Fib across different TNM stages in the ScRNA.GSE178341 cohorts.

**Supplementary Figure 4. Presence and Enrichment of Col10a1⁺ Fibroblasts in Mouse Models.**

A. UMAP plot of 28,604 single cells from the analysis, grouped by cell type. Each cell type is color-coded and labeled in the legend.

B. Dot plot showing the expression of selected marker genes across different cell types. The size of each dot indicates the percentage of cells expressing the gene, while the color represents the z-score of the gene expression level, ranging from -1 (low expression) to +1 (high expression).

C. t-SNE plot of 1,163 fibroblast cells, colored by their identified Seurat clusters (0 to 5). Each cluster is represented by a different color and contains the corresponding number of cells.

D. Left: t-SNE plot showing the expression density of the Col10a1 gene across the fibroblast clusters. The density of Col10a1 expression is indicated by a color gradient. Right: Violin plot showing the expression level of Col10a1 across the identified fibroblast clusters, with statistical significance indicated by asterisks.

***P < 0.001; ****P < 0.0001.

**Supplementary Figure 5. Functional Characterization of COL10A1⁺Fib in Promoting Metastasis and Immunosuppression.**

A. GSVA analysis of COL10A1⁺Fib functional pathways (HALLMARK) in the Merge.ScRNA cohort.

B. Overlapping differentially expressed genes in COL10A1⁺Fib between Merge.ScRNA and ScRNA.GSE178341 datasets.

C. GSEA enrichment analysis (GO, KEGG, HALLMARK) based on COL10A1⁺Fib differentially expressed genes.

D–E. GSVA enrichment analysis of hallmark pathways in patients with high vs. low COL10A1⁺Fib infiltration in the TCGA and Bulk.GEO.Merge datasets.

F. Single-cell RNA-seq analysis of GEO.LM dataset (n = 30,449 cells). Left panel: UMAP plot of single cells from various cell types. Middle panel: Dot plot showing the expression of key marker genes across different cell types, with the size of each dot representing the percentage of cells expressing the gene and the color indicating the z-score of gene expression levels (ranging from -1 to +1). Right panel: t-SNE plot of 496 fibroblast cells from GEO.LM dataset, color-coded by cell subclusters (LM.C1, LM.C2, LM.C3, LM.C4).

G. Expression of COL10A1 in fibroblast clusters (LM.C1, LM.C2, LM.C3, and LM.C4). Left panel: t-SNE plot showing the density of COL10A1 expression in fibroblast subclusters, colored by expression density. Right panel: Violin plot showing COL10A1 expression levels across fibroblast subclusters (LM.C1, LM.C2, LM.C3, LM.C4), with statistical significance indicated by asterisks.

H. GSVA of different fibroblast subsets.

I. COL10A1⁺Fib signature score across the consensus molecular subtypes (CMS) of CRC；CMS1 (n=14), CMS2 (n=23), CMS3 (n=15), CMS4 (n=28).

**P < 0.01; ***P < 0.001; ****P < 0.0001.

**Supplementary Figure 6. Identification and Spatial Mapping of Cell Subpopulations Adjacent to COL10A1⁺Fib.**

A. Re-clustering results of six major non-fibroblast lineages, including 10 epithelial, 7 myeloid, 9 T/NK, 6 endothelial, 7 plasma, and 6 B cell subclusters.

B. Representative spatial mapping of all 56 subclusters based on integration of ST data with Merge.ScRNA reference.

C. Spatial distance matrix between COL10A1⁺Fib and the remaining 55 subpopulations.

**Supplementary Figure 7. COL10A1 Signaling Network Analysis.**

A. COL10A1 signaling circle plot showing intercellular communication. Nodes represent subclusters; red lines indicate outgoing signals from COL10A1⁺Fib, with line thickness reflecting communication strength.

B. Heatmap of COL10A1 signaling probabilities. Rows represent sender subpopulations and columns represent receivers; color intensity indicates communication probability.

C. Role heatmap depicting the functional roles of each cell subpopulation in COL10A1 signaling (Sender, Receiver, Mediator, Influencer), with green shading denoting importance scores in the signaling network.

**Supplementary Figure 8. Characterization of Epi_1 epithelial subpopulation adjacent to COL10A1⁺Fib.**

A. Proportion of Epi_1 cells in normal vs. tumor tissues and across different TNM stages in the Merge.ScRNA dataset.

B. GSVA heatmap showing pathway enrichment in the Epi_1 cluster.

C. Kaplan–Meier survival analysis (RFS and OS) for patients with high vs. low Epi_1 abundance in TCGA and Bulk.GEO.Merge cohorts.

D. Schematic illustration of copy number variation (CNV) analysis at the single-cell level.

E. tSNE plot of CNV scores and CNV differences among epithelial subclusters.

F. Epi_1 epithelial cells exhibit high expression of ERO1A and ALDOA, along with other top marker genes.

**Supplementary Figure 9. In vitro functional validation of COL10A1⁺Fib and EMT phenotype analysis.**

A. Construction of the COL10A1⁺Fib model: Western blot shows COL10A1 over-expression (OE) compared with the empty-vector control (OE_NC); quantification is presented in the right-hand panel; Cell line used: CAFs.
B. Construction of the COL10A1⁻ Fib model: Western blot analysis demonstrated that CRISPR knockdown with three independent sgRNAs (Sg_1–3) markedly reduced COL10A1 expression versus the non-targeting control (Sg_NC); Sg_1 was chosen to establish the COL10A1⁻ Fib line. Quantification appears in the right-hand panel; Cell line used: CAFs.

C–D. CCK-8 and colony formation assays validating the effects of COL10A1⁺Fib in the SW620 CRC cell line.

E-F. Tumor volume and tumor weight measurements in xenograft-bearing mice across experimental groups.

G. Representative histological assessment of xenograft tumors from the four experimental groups (per group: n=6); Upper row: H&E staining; Lower row: Ki67 immunohistochemistry; Scale bar = 200 μm.

H. Migration and invasion assays (Transwell) confirming COL10A1⁺Fib-mediated enhancement of SW620 motility. Scale bars, 100 μm.

I. Immunofluorescence analysis of EMT markers (E-cadherin, vimentin) in HCT116 and SW620 cells after co-culture with COL10A1⁺Fib. Scale bars, 50 μm.

***P < 0.001.

**Supplementary Figure 10. Expression of macrophage-subtype markers and spatial co-localisation of COL10A1⁺ fibroblasts with M2 macrophages.**

A. Violin plots from the GEO.Merge.ScRNA dataset illustrate the expression levels of the macrophage markers APOE, SPP1, and C1QA. ****P < 0.0001.
B. Spatial-transcriptomic analysis demonstrates a strong spatial overlap between COL10A1⁺ fibroblasts and M2 macrophages within colorectal-cancer tissue.

**Supplementary Figure 11. Additional data supporting COL10A1/CD18-mediated signaling in M2 polarization.**

A. Western blot comparison of CD18 protein expression among M0, M1, and M2 macrophages.

B. Western blot validation of CD18 expression changes in M0 macrophages under different treatments.

C. Predicted COL10A1–CD18 docking models visualizing the top 10 docking poses; CD18 is shown in a single solid colour; COL10A1 is rainbow-coloured from N-terminus (blue) to C-terminus (red).

D. Confidence scores for the top 10 COL10A1–CD18 docking models.

E. Western blot validating siRNA knockdown efficiency of CD18.

**P < 0.01; ***P < 0.001.

**Supplementary Figure 12. TGF-β signaling from M2-like macrophages regulates COL10A1⁺Fib.**

A. Dot plot of ligand–receptor interactions from cell communication analysis showing dominant TGF-β ligand–receptor pairs (e.g., TGFB1-ACVR1+TGFBR1, TGFB1-TGFBR2+TGFBR1, TGFB1-ACVR1C+TGFBR2) between M2 macrophages and COL10A1⁺Fib.

B. Network diagram of TGF-β signaling intensity and direction from M2 macrophages to COL10A1⁺Fib and other fibroblast subtypes.

C. Heatmap of communication probabilities in the TGF-β pathway between cell types, highlighting M2 macrophages to COL10A1⁺Fib as the strongest interaction.

**Supplementary Figure 13. Identification and validation of NU7441 as an inhibitor of COL10A1⁺Fib function.**

A. Volcano plots showing correlations between IC50 values of 198 drugs and COL10A1⁺Fib infiltration in TCGA and Bulk.GEO.Merge cohorts.

B. Venn diagram of 10 drugs negatively correlated with COL10A1⁺Fib infiltration in both datasets.

C. Scatter plots showing correlation of the 10 candidate drugs with COL10A1⁺Fib infiltration scores.

D. Molecular docking models of the 10 candidate compounds with COL10A1 protein, including Vina scores.

**Supplementary Figure 14. Effect of NU7441 on TGF-β1, RUNX2, and CD18/JAK1/STAT3 Signaling Pathway, and Molecular Dynamics Analysis of the COL10A1–NU7441 Complex.**

A. Western blot analysis showing effect of NU7441 on TGFB1 expression in COL10A1⁺Fib.
B. Western blot analysis showing reduced expression of RUNX2 in COL10A1⁺Fib following treatment with NU7441.
C. Western blot and quantification of CD18/JAK1/STAT3 signaling pathway expression in M0 macrophages across groups.

D. RMSD trajectory comparison for COL10A1, NU7441, and COL10A1–NU7441 complex.

E. Changes in the number of hydrogen bonds formed between NU7441 and COL10A1.

F. RMSF analysis showing flexibility of COL10A1 residues before and after binding NU7441.

G. Rg plot illustrating the compactness of the COL10A1–NU7441 complex.

H. SASA plot showing solvent exposure of the complex during simulation.

**Supplementary Figure 15. COL10A1 Expression and Functional Characteristics in Bulk and Single-Cell Pan-Cancer Datasets.**

A. Differential expression analysis of COL10A1 between tumor and normal tissues across nine cancer types (bulk RNA-seq data).

B. Kaplan–Meier survival curves evaluating the association between COL10A1 expression and overall survival (OS).

C. Heatmap of representative gene expression across different cancer types and cell lineages in scRNA-seq datasets.

D. t-SNE projections of scRNA-seq data from nine tumor types, colored by dataset origin.

E. Violin plots comparing COL10A1 expression between tumor and normal tissues across individual cancer types.

*P < 0.05; ***P < 0.001; ****P < 0.0001.

**Supplementary Figure 16. Spatial Distribution of COL10A1⁺Fib Across Eight Tumor Types**

**Spatial co-distribution maps showing overlap between COL10A1⁺Fib, epithelial–mesenchymal transition (EMT) signatures, M2 macrophage infiltration, and high-density tumor cell regions across eight cancer types (lung, liver, gastric, breast, pancreatic, prostate, cervical, and ovarian cancers).**
